# Management of psychotropic medications in adults with intellectual disability: a scoping review

**DOI:** 10.1080/07853890.2022.2121853

**Published:** 2022-09-18

**Authors:** Ashley Costello, Eithne Hudson, Susan Morrissey, Drona Sharma, Dervla Kelly, Owen Doody

**Affiliations:** a School of Medicine, University of Limerick, Limerick, Ireland; b Intellectual Disabilities, Nua Healthcare Services, Naas, Ireland; c Health Research Institute, University of Limerick, Limerick, Ireland; d Department of Nursing and Midwifery, University of Limerick, Limerick, Ireland

**Keywords:** Behaviours that challenge, intellectual disability, medication management, mental health, polypharmacy, psychotropics, scoping review

## Abstract

**Background/objective(s):**

Psychotropic medications are commonly prescribed among adults with intellectual disability, often in the absence of a psychiatric diagnosis. The aim of this scoping review is to provide an overview of the extent, range, and nature of the available research on medication use and practices and medication management in people with intellectual disability taking psychotropic medications for behaviours that challenge.

**Materials and methods:**

A scoping review of research studies (qualitative, quantitative, and mixed design) and Grey Literature (English) was carried out. Databases included: Ovid MEDLINE, Embase, CINAHL, JBI Evidence Synthesis, Cochrane Central Register of Controlled Trials, Cochrane Database of Systematic Reviews, PsycINFO, and Scopus. A three-step search strategy was followed, with results screened by two independent reviewers. Data was extracted independently by two reviewers using a data extraction tool with results mapped and presented using a narrative form supported by tables and diagrams to the research questions.

**Results:**

Following the removal of duplicates, records were screened, full texts assessed, and 49 studies were included. Medication outcomes included reduced repetitive, stereotypic, and/or aggressive behaviours. High dosing/prescribing in the setting of an absent/unclear clinical indication was associated with worsening of symptoms for which psychotropics were prescribed. While psychotropics had a role in managing behaviours that challenge, reducing or discontinuing psychotropics is sometimes warranted. Study designs were frequently pragmatic resulting in small sample sizes and heterogeneous cohorts receiving different doses and combinations of medications. Access to multidisciplinary teams, guidelines, medication reviews, staff training, and enhanced roles for carers in decision-making were warranted to optimize psychotropic use.

**Conclusions:**

These findings can inform prescribing interventions and highlight the need for timely and comprehensive patient outcome data, especially on long-term use of high doses of psychotropics and what happens when reduce or stop prescribing these doses.

**KEY MESSAGES**
- Psychotropic medications are frequently prescribed for people with intellectual disabilities, often at high doses and these medications are associated with both positive and negative patient outcomes.
- Work to rationalize psychotropic use has been reported with interventions aiming to reduce polypharmacy or deprescribe a single psychotropic medicine. These interventions had mixed success and risk of relapse was documented in some studies.
- Limitations in sample size and heterogenous patient cohorts make it challenging to understand the risks and benefits associated with reducing or stopping psychotropic medicines.
- Patient, carer, and clinician partnerships are critical to advance medication management.

## Introduction

Intellectual disability (ID) is characterized by impaired intellectual functioning and learned behaviour, which affects various everyday social and practical competencies [1]. Psychotropic medications are commonly prescribed amongst people with ID and include anti-psychotics, anti-depressants, mood stabilizers including anti-epileptic medications, anti-anxiety medications including benzodiazepines, psychostimulants, beta-adrenergic blockers, z-drugs/sleeping tablets and opioid antagonists. Some of these medicines are sometimes referred to as hypnotics or sedatives. The reported rates of use of these medications vary between 32 and 85% and are thought to be higher than in the general population [2,3].

Uncertainties have been raised regarding the use of psychotropic medications amongst the ID population as they are associated with a multitude of risks and side effects, potential ineffectiveness, and long-term use without review [4,5]. The Winterbourne View abuse scandal criticized the inappropriate use of psychotropic drugs [6]. There is a weak correlation between the prescription rates of these medications and the rates of diagnosed mental disorder [7,8]. For instance, an Irish ID cohort study on ageing reported that 45.1% of the study population were taking anti-psychotics, and of those 25.9% had a diagnosed psychiatric illness [7]. This incongruity has been attributed to the “off-label” use of psychotropic medication for the management of behaviours that challenge. A diagnosis of psychiatric illness in adults with ID is determined by the severity of the ID, the cause of the ID, psychiatric disorders including developmental disorders, psychiatric illness, personality disorders, problem behaviours, and others [9]. The Royal College of Psychiatrists defines behaviours that challenge as: “a behaviour of such an intensity, frequency or duration as to threaten the quality of life and/or the physical safety of the individual or others and is likely to lead to responses that are restrictive, aversive or result in exclusion” [10].

Behaviours that challenge frequently recur and those who are started on psychotropic medicines tend to continue on them for prolonged durations. Risks associated with long-term psychotropic use include movement (extra-pyramidal) side effects, involuntary autonomic disturbances, and endocrine and metabolic disorders [11]. Some can cause apathy and those with a high level of anti-cholinergic activity might diminish attention and reasoning, especially when used with other anti-cholinergic drugs [12]. While several UK guidelines exist (e.g. NICE guidelines, Frith Prescribing Guidelines, STOMP) recommending psychological and environmental interventions as the initial treatment for the management of behaviours that challenge, there is a poor implementation in practice [13–15]. In addition, people with ID tend to be treated for long periods of time with prescriptions often remaining unchanged despite the associated risks.

The aim of this scoping review is to provide an overview of the existing research on the use of psychotropic medications in adults with ID to manage behaviours that challenge them. Although reviews have been undertaken previously [16,17], this review intends to give an up-to-date review of the published evidence. Specifically, this review plans to scope and map what psychotropic medications are prescribed to adults with ID, any indications for the prescription, safety concerns, and professional and lay strategies that exist to manage these medications over the long term. This will include studies that monitor medication side effects and the impact of reducing or stopping psychotropic medications. Any psychological or social educational intervention components for behaviours that challenge running side by side with medication-related interventions will also be reviewed and mapped. This scoping review will help in identifying safety issues associated with psychotropics and reactive practices and help inform future research and practice in this area.

## Methods

**Figure 1.**
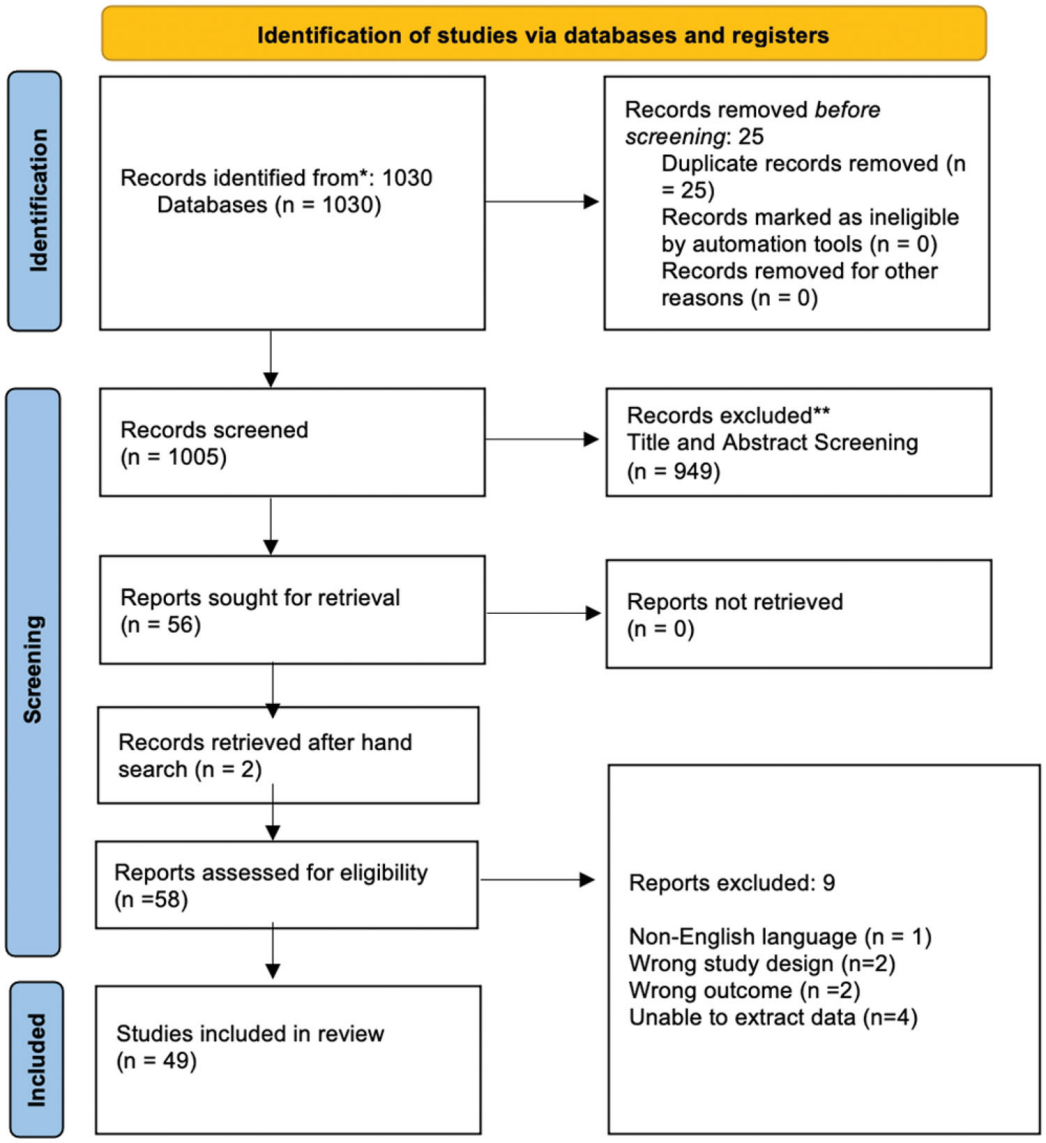
PRISMA flow diagram. From Page et al. [26]. For more information, visit: http://www.prisma-statement.org/.

Arksey and O’Malley [18] six-stage framework and the Preferred Reporting Items for Systematic Reviews and Meta-Analyses Extension for Scoping Reviews [19] were utilized in the conduct and reporting of this review. Before conducting the review, the protocol was prepared and published in HRBOpen [20]. Scoping review developments by Levac et al. [21], Peters et al. [22], Bradbury-Jones et al. [23], and Westphaln et al. [24] were incorporated along with the PRISMA flow chart [25] (Figure 1). In stage one, when identifying the research question, the focus was to map the existing literature on psychotropic medication use by people with ID which was addressed by the sub-questions:

(Q1) What psychotropic medications are commonly prescribed among adults with ID?
(Q2) What is the clinical indication(s) for prescription of such medications?
(Q3) What evidence base (if any) exists to support the prescription of psychotropic medications, including “off-label” use in adults with ID?
(Q4) What guidelines/policies/frameworks or practices exist regarding the management of psychotropic medicines once they are prescribed to people with ID?
(Q5) What interventions (if any) are available to facilitate dose reduction or cessation of psychotropic medications among people with ID?
  – How have such interventions been evaluated to date? i.e. what outcomes are measured?
  – What are the potential benefits and risks associated with the reduction or cessation of psychotropic medication?

In stage two, searching for relevant studies was guided by the PCC process (participant, concept, context). “Participants” were adults over 18** **years with ID, psychotropic medications was the “Concept” and reporting use, safety, behaviour, professional practices in managing psychotropic medications were the “Context”. Both database and grey literature searches were performed on the following: CINAHL, PsycINFO, Medline Ovid, Embase, JBI Evidence Synthesis, Cochrane Central Register of Controlled Trials, Cochrane Database of Systematic Reviews and Grey literature databases (Open Grey, ETHOS, ProQuest) using keywords/terms with Boolean operators (Supplementary File 1). All results were exported to the Endnote reference manager, where duplicates were removed. Screening and voting were carried out using Rayyan.

In stage three, when selecting studies, independent screening and voting were carried out by two reviewers against the inclusion criteria presented in Table 1. Initial screening was by title and abstract, and then at full text. Conflicts were resolved by involving a third reviewer or consensus. In stage four, when charting the data, data were extracted from all studies meeting the inclusion criteria into a predetermined data extraction table (Table 2). The table addressed details pertaining to authors, year and country, medication usage (Q1, Q2), effectiveness (Q3), management and outcomes (Q4, Q5). In stage five, the extracted data were charted and reported descriptively with results mapped and presented in relation to the review questions. A PRISMA flow chart was completed to demonstrate study eligibility, screening, selection, and number of included studies. The results were presented in descriptive form using the sub-questions as headings and appropriate tables and diagrams were used/developed to illustrate the findings and enhanced by narrative text. We did not formally assess the methodological quality of the identified studies due to anticipated heterogeneity in study types and designs [22]. In line with the objective of scoping reviews the results assisted in making recommendations.

**Table 1.** Inclusion exclusion criteria.

Inclusion criteria	Study design: all research designs including reviews (systematic, integrative, and narrative) and research (qualitative, quantitative, and mixed design studies). In addition, national and international policies, strategies, guidelines, and standards will also be examined. Year of publication: No restriction. Language: English language only.
Exclusion criteria	Article types: commentaries, editorials, opinion pieces, non-systematic literature reviews, case studies. Clinical trials of medicinal product.

**Table 2.** A summary of 15 studies that reported prevalence of psychotropic medication use.

References	Prevalence of psychotropic medication prescribed	Study setting
Branford [27]	49% (*n*** **=** **98) thioridazine, 26% (*n*** **=** **52) chlorpromazine, 17% (*n*** **=** **34) zuclopenthixol, and 8% (*n*** **=** **15) haloperidol of a sample of 198.	In-patient and community settings.
Bowring et al. [28]	37.73% (*n*** **=** **100) of a sample of 265.	In-patient and community settings.
de Kuijper and Hoekstra [29]	9% (*n*** **=** **88) of a sample of 88 prescribed 2 or more psychotropics.	Community setting.
Deb et al. [2]	88% (*n*** **=** **88) of a sample of 100 at baseline (T1). 91% (*n*** **=** **70) of a sample of 77 at 6** **month follow up (T2).	Community setting.
Erickson [30]	86.7% (*n*** **=** **13) of a sample of 15.	Community setting.
Espadas et al. [31]	48% (*n*** **=** **40) antipsychotics, 22% (*n*** **=** **18) anticonvulsants, 17% (*n*** **=** **14) anxiolytics, and 13% (*n*** **=** **11) antidepressant all of a sample of 83.	Community setting.
Holden and Gitlesen [32]	110 (37.4%) of a sample of 300.	Community setting.
Kastner et al. [33]	88.9% (*n*** **=** **16) of a sample of 18.	Tertiary-care setting.
Niven et al. [34]	58% (*n*** **=** **61) of a sample of 106.	Community setting.
Perry et al. [35]	90% (*n*** **=** **322) of a sample of 358.	In-patient and community settings.
Sachdev [36]	60.4% (*n*** **=** **32) of a sample of 53.	Inpatient setting.
Song et al. [37]	43% (*n*** **=** **39) of a sample of 138 in 2002, 51% (*n*** **=** **42) of a sample of 93 in 2006, 53% (*n*** **=** **57) of a sample of 111 in 2007–2011, and 54% (*n*** **=** **45) 2012–2015 of a sample of 92.	Community setting.
Tan et al. [38]	Antidepressants 71.2% (*n*** **=** **2160), Antipsychotics 58.4% (*n*** **=** **1772), Anxiolytics, sedatives, hypnotics 32.0% (*n*** **=** **967), CNS stimulants 3.8% (*n*** **=** **116), Miscellaneous CNS agents 0.7% (*n*** **=** **22) all in a sample of 3033.	Community setting.
Tsakanikos et al. [39]	76.8% (*n*** **=** **52) of a sample of 69.	Community setting.

There were some modifications to the protocol: Research question 4 has been expanded from mapping existing medication guidelines alone to also including frameworks and practices. This is to reflect professional and non-professional medicine management studies found in the literature [20].

Supplementary File search strategy (example of one).

## Results

Following the removal of duplicates, 1005 records were screened, and 57 full-text records were assessed for inclusion (Figure 1). Ultimately 48 studies were included in the review (Supplementary Data Extraction Table 3). Most studies were undertaken in a single country (*n*** **=** **45); USA (*n*** **=** **13), UK (*n*** **=** **13), the Netherlands (*n*** **=** **5), Germany (*n*** **=** **4), Canada (*n*** **=** **2), Australia (*n*** **=** **2). Croatia, Ireland, Italy, Norway, New Zealand, and Spain had one study, respectively. Three studies were undertaken in more than one country (UK and Australia *n*** **=** **1; UK, China, Sri Lanka *n*** **=** **1; UK and Jersey *n*** **=** **1) (Supplementary Data Extraction Table 3).

Of the 48 included studies, they mapped to the research questions as follows:

(RQ1) Description of commonly prescribed psychotropic medicines among adults with ID: 13 studies.
(RQ2) Clinical indication(s) for prescription of such medications? 26 studies.
(Q3) What evidence base (if any) exists to support the prescription of psychotropic medications, including “off-label” use in adults with ID? 16 studies.
(Q4) What guidelines/policies or practices exist regarding the management of psychotropic medicines once they are prescribed among people with ID? 11 studies.
(Q5) What interventions (if any) are available to facilitate dose reduction or cessation of psychotropic medications among people with ID? 16 studies.

### RQ1: What psychotropic medications are commonly prescribed among adults with ID?

The reported prevalence of psychotropic medication use in adults with ID across the studies ranged between 37.4 and 91.0% (Table 2), with studies set in a variety of context(s), such as inpatient, community, and tertiary care settings. Antipsychotic medications were reported as the most commonly prescribed psychotropic medications with prescribed rates ranging from 12.6 to 79.0% across various studies [2,27–39]. There was evidence that the type of antipsychotic medications changed over time, where older studies reported first-generation antipsychotic medications being prescribed more often, such as thiorazidine, chlorpromazine, zuclopenthixol, and haloperidol [27,32]. While more recent studies report next-generation antipsychotic medications, in particular risperidone, olanzapine and quetiapine as being the most prescribed antipsychotics [28,31].

While antipsychotic medications were overall the most prescribed psychotropic medication, a large USA-based study [38] identified antidepressant medications as the most commonly prescribed with a rate of 71.2% or 2160 participants of a sample of 3033. However, other studies identified lower rates ranging from 2.7 to 21% [28,30–32]. The prescribed rate of anticonvulsant medications ranged between 5.1 and 43% [30,32,37–39]. Notably, anxiolytics, sedative, and hypnotic medications accounted for almost one-third of psychotropic medications prescribed at 31.88% or 967 participants of a sample of 3033 [38]. Sedative or hypnotic medications including melatonin were reported at 3.8% [30] and 4.3%, and anxiolytic medications alone prescribed rates ranged from 1.7 to 35% [30–32,34]. Of concern was that polypharmacy of multiple prescriptions of psychotropic medications was identified across numerous studies [2,28,31,32,36] and often involved multiple psychotropic medications [2,27].

### RQ2: What is the clinical indication(s) for prescription of psychotropic medications?

Clinical indications for the prescription of psychotropic medication for individuals with ID were often unclear. Indications, such as “psychiatric disorder” and “problem behaviours” were recognized within the literature, without further elaboration as to specific diagnoses/detailing of symptoms [32,40,41]. Furthermore, prescription of psychotropic medication within this population may occur where there is no clear clinical indication and in the absence of a documented behavioural/emotional disturbance or psychiatric diagnosis [32,37,41]. One study noted that prescriber discipline (psychiatry *vs.* GP) may also influence whether a clear clinical indication is noted [32].

Where specific clinical indications were noted, these were categorized into behavioural and psychiatric conditions/symptoms. Behavioural indications consisted of: behaviours that challenge, stereotypy, aggression, emotional disturbance, destructive and disruptive behaviour, self-injury, restlessness, irritability, hyperactivity, and behavioural issues associated with an autism spectrum disorder or pervasive developmental disorder [2,27–29,37,39,41–44]. One study found behaviours that challenge was a predictor of psychotropic medication use after controlling for other variables; data indicated that there may be differences in prescribing patterns associated with different types of behaviours that challenge (e.g. total challenging behaviour, aggressive and destructive behaviour, self-injury and stereotypy) [28]. Psychiatric indications included: non-schizophrenia related psychotic symptoms, chronic psychotic disorder and bipolar disorder [29,37,44,45]. Antipsychotic medications were used primarily for managing behavioural or emotional disturbances, rather than for the treatment of psychiatric disorders [41,45].

Where diagnoses/symptoms/problem behaviours alone and combination were equally represented as indications; the most common medication types were antipsychotics, followed by anticonvulsants [32,45]. Divalproex and valproate were indicated for the treatment of self-injurious, aggressive, or destructive behaviour, particularly in patients with coexisting epilepsy [33,44]. Zuclopenthixol, a first-generation antipsychotic, may also be clinically indicated for exacerbations of aggressive behaviour; a trial in individuals with mild to moderate ID demonstrated an increase in aggressive behaviour when it was withdrawn [43].

Additionally, valproate was considered the treatment of choice for bipolar disorder while newer, atypical antipsychotics (particularly risperidone) were indicated for self-injurious behaviour [44]. New generation antipsychotic medications have a role in treating complex cases, favoured as they are considered to have less side effects than clozapine and first-generation antipsychotic medications and produce less pyramidal symptoms [11]. However, metabolic syndrome complications may be more evident with some new generation antipsychotic medications when compared with conventional antipsychotic medications and they are notably more expensive which may be a consideration [11].

### RQ3: What evidence base exists to support the prescription of psychotropic medications, including “off-label” use in adults with ID?

The 15 studies that mapped to this question reflected varying types of evidence for the use of psychotropic medications within this population (Table 3). Three studies had a prospective cohort design [2,36,46] and three studies were retrospective [47–49]. A wide variety of trial designs were evident, encompassing pragmatic trials [33,50,51] to randomized placebo-controlled trials [52,53]. Four review articles were also included [11,42,54,55]. This section describes studies reporting the effectiveness of psychotropic medication to reduce behavioural and/or psychiatric symptoms and reporting of adverse effects; with discontinuation or reduction of psychotropic medication explored in research question 5.

**Table 3.** A summary of 15 studies that reported efficacy or side effects of psychotropic medication use in people with ID.

Study design		Key findings
Prospective cohort study	Deb et al. [2]	Risperidone, chlorpromazine, haloperidol, olanzapine, zuclopenthixol, quetiapine, SSRIs (citalopram, paroxetine, and fluoxetine), and mood stabilizers (carbamazepine and sodium valproate) in higher doses—more severe aggressive behaviour, physical aggression towards objects, self-injurious behaviour
	Drmic and Franic [46]	Olanzapine—improvement in disruptive behaviour
	Sachdev [36]	Neuroleptics—associated with tardive dyskinesia
Retrospective	Janowsky et al. [47]	Reduction of Haloperidol or Haloperidol equivalents in the case of Thiothixene and Loxapine—all relapses were clinically significant
	Ruedrich et al. [48]	Divalproex sodium or valproic acid—Improvement in self-injurious behaviour/disruptive behaviour
	Ruedrich et al. [49]	Risperidone, quetiapine, olanzapine—decreased aggression
Pragmatic trial with variety of design	Kastner et al. [33]	Valproic acid—improvement in problem behaviour
	Troisi et al. [50]	Fluoxetine—increased aggression
	Tyrer et al. [51]	Placebo, haloperidol, and risperidone—reduction in aggression was noted with all treatments after 4** **weeks (greatest decrease was with placebo)
Randomized controlled trial	Schwarz et al. [52]	Zuclopenthixol—decreased aggression
	Hässler et al. [53]	Zuclopenthixol—improvement in disruptive behaviour
Systematic/narrative review	Aman and Singh [42]	Antipsychotic drugs—reduced adaptive behaviour and learning
	Ji and Findling [54]	Methylphenidate—reduction in ADHD symptoms Lithium—reduced aggression Antidepressants—sometimes poorly tolerated with limited evidence of efficacy
	de Leon et al. [11]	NGA drugs—less toxic than clozapine, less EPS. Metabolic syndrome complications may be worse with some NGAs *vs.* high-potency conventional antipsychotics.
	Sohanpal et al. [55]	Antidepressants, particularly SSRIs, improve aggression, SIB, and other behaviour problems on average in <50% of cases, and the rest show either no improvement or deterioration

NGA: next-generation antipsychotics; EPS: extrapyramidal side effects.

Sample sizes ranged from 19 people [50] to 100 people [2]. Data collection mostly took place in community settings [2,46–49,51–53], with one study taking place in tertiary care [33] and two in an inpatient setting [36,50]. Studies employed specific scales to assess desired outcome measures with a variety of scales seen across studies. Commonly utilized scales were the Aberrant Behaviour Checklist (ABC) [2] and its subscales, the Modified Overt Aggression Scale (MOAS) [2,50–53], the Disability Assessment Schedule (DAS) [53], and the Clinical Global Impression Scale and its subscales (CGI-S) [33,46,53].

Some studies demonstrated short- and long-term improvements in problem behaviours using olanzapine [46,49], zuclopenthixol [52,53], valproic acid and divalproex sodium [33,48]. A trial comparing haloperidol, risperidone, and placebo for aggression demonstrated that all treatments reduced aggression, with placebo associated with the greatest reduction [51]. Conversely, fluoxetine was associated with an increase in aggressive behaviour [50]. An earlier review reported limited evidence to support the use of antiepileptic, anxiolytic, and naltrexone medications for the management of problem behaviours [54]. Antidepressants were found to be poorly tolerated and ineffective in reducing repetitive/stereotypic behaviour in several studies [2,50,54,55]. Side effects have also been reported for antipsychotic medications including severe aggressive behaviour, physical aggression towards objects, self-injurious behaviour [2], and reduced adaptive behaviour and learning [42].

### RQ4: What guidelines/policies or practices exist regarding the management of psychotropic medicines once they are prescribed among people with ID?

Ten papers addressed professional or lay practices in relation to managing psychotropic medications: two papers produced guidelines for prescribers [11,56], one paper reviewed pharmacists' role in the management of medications [57], three studies used surveys [29,40,44], three studies used qualitative interviews [58–60] and one study used mixed methods [41] to explore stakeholder views of using psychotropic medications and what influences their decision making.

In relation to the guidelines, Sabaawi et al. [56] present guidelines for the use of clozapine in individuals with developmental disabilities. They encouraged prescribing psychiatrists to make a clinical judgemental on the use of clozapine in individuals with ID on a case-by-case basis; consent to periodic venipuncture for monitoring purposes is required; and identified clinical indications including DSM-IV-TR diagnosis of schizophrenia or schizoaffective disorder that failed to tolerate or respond to previous treatment or severe persistent or self-injurious behaviour with evidence that a behavioural treatment was ineffective. The guidelines acknowledge the lack of evidence available regarding the use of clozapine for self-injurious behaviour but state that a 3-month trial of clozapine at a sustained plasma concentration of at least 350** **ng/ml is a reasonable approach. These guidelines also offer guidance on how to monitor for side effects once initiated which includes weekly full blood counts, weight, serum glucose, and lipids monitoring.

de Leon et al. [11] provide practical guidelines for the use of new generation antipsychotic medications (except clozapine) in adult individuals with ID. They recommend regular weight fasting blood glucose, serum lipids, tardive dyskinesia rating, serum prolactin, and a breast examination be carried out [11]. An annual waist circumference and ECG should also be included and an eye examination for those taking quetiapine [11]. These guidelines also encourage physicians to be vigilant about the development of potentially lethal complications of new generation antipsychotic drug use including neuroleptic malignant syndrome, diabetic coma, pancreatitis, and the risk of arrhythmias [11].

Eight papers referenced the NICE guidelines in the introduction and/or discussion and often in support of the rationale for the research [2,17,28,35,37,45,61,62]. The NICE guidelines emphasize that antipsychotic medications should not be used to treat problem behaviour unless other non‐pharmacological approaches have been tried and failed and the person with ID or others are at serious risk of harm. The Royal College of Psychiatrists in the UK [63] document surrounding psychotropic medication prescribing in ID was also cited [35]. One study noted the absence of guidelines for the pharmacological management of psychiatric symptoms in older adults with ID [64].

Seven studies described other health systems, professional and carer factors that influence medication-related decisions in practice [29,40,41,44,58–60]. Sheehan et al. [60] emphasize the role of family and paid carers in undertaking several medication-related activities, such as collecting, storing, and giving medication, monitoring health, and advising a person on when and where to seek professional advice. These carers felt they had important information useful to inform decisions about medicines although sometimes being insufficiently included in the discussion and lacking influence were sometimes barriers [60]. A second study found the attitude of carers towards medicines has implications for medicine compliance and stigma about the use of psychiatric medications use can result in a negative view towards taking these medications [59].

A Canadian study surveying clinical staff identified a lack of specialists with training in ID and access to services dependent on individual financial resources and insurance [40]. Another study in the Netherlands found psychotropic medication prescribing depended on the preferences of prescribers who were aware of the guidelines but developed their own from experience and felt medications were sometimes the only treatment option for behavioural and psychiatric issues in people with ID [58]. Similarly, another study noted variation in prescribing which may be related to culture or organization factors [29]. Patel et al. [44] surveyed psychiatrists and medication experts about their opinions on the use of psychotropic medications for mental illness in patients with ID and found they were broadly in agreement. The practitioners rated venlafaxine and mirtazapine higher than the medication experts [44]. Lithium augmentation of therapy with selective serotonin-reuptake inhibitors for nonpsychotic depression was rated first-line by the practitioners and second-line by the medication experts [44]. A mixed-methods study comprising of focus groups and a survey showed that a large majority of support staff perceived antipsychotic medications to be effective at controlling behaviours that challenge, despite the lack of scientific evidence [41]. The staff also emphasized the need to balance the benefits and side effects of these medications [41]. Blunted affect is perceived as the side effect with the most impact, which, in the perception of support staff, these side effects are often caused by a too high dosage of antipsychotic medications [41].

### RQ5: What interventions (if any) are available to facilitate dose reduction or cessation of psychotropic medications among people with ID?

– **How have such interventions been evaluated to date? i.e. what outcomes are measured?**
– **What are the potential benefits and risks associated with the reduction or cessation of psychotropic medication?**

Fourteen studies describing dose reduction or cessation of psychotropic medications were identified [2,17,41,43,61,65–73] with one of these being a systematic review [17]. Two papers produced medication review tools for use by healthcare professionals [62,74].

Discontinuation/reduction studies demonstrated a mix of improvement and deterioration in psychiatric symptoms/problem behaviours and metabolic adverse effects [43,52,61,67,68]. A controlled discontinuation study of long-term antipsychotic medications prescribed for behavioural disturbances demonstrated both statistically and clinically significant reductions in weight, waist circumference, Body Mass index (BMI), and systolic blood pressure by 3.5** **kg, 4** **cm, 1.4** **kg/m^2^, and 7.1** **mmHg, respectively (*N*** **=** **99). Interestingly, a decrease in weight and BMI was reported even in participants who did not achieve full discontinuation, highlighting the potential health benefits associated with the reduction and discontinuation of antipsychotic medications [67]. Hanzel et al. carried out a retrospective review of the records of adults with a dual diagnosis of ID and epilepsy who were on an established regimen of phenobarbital (barbiturate antiepileptic drug (AED) and antipsychotic medication. Phenobarbital was gradually reduced over a two-year period and replaced with another AED: carbamazepine or valproic acid. The authors reported a reduction in behaviours that challenge by 81.5% following discontinuation of phenobarbital, with complete discontinuation achieved in two cases [68]. Conversely, discontinuation of zuclopenthixol was associated with symptom deterioration [69]. It was found that the placebo subgroup where zuclopenthixol was withdrawn (*n*** **=** **20) exhibited more aggressive behaviour than the continuing subgroup (*n*** **=** **19), suggesting that discontinuation of Zuclopenthixol in this population leads to an increase in aggressive behaviour [43]. At 2-year follow-up, the patient group that had continued zuclopenthixol treatment showed significant benefits across all efficacy measures (DAS, MOAS, CGI) while the group who had discontinued treatment displayed no improvement across any efficacy measure, demonstrating that zuclopenthixol is significantly superior to placebo for maintenance of aggressive behaviour in adults with ID [53].

Withdrawal success rates were sometimes reported: 7% [70] 25% [66], 33% [65], 43% [67], and 46.5% [61], with inconsistencies explained by partially withdrawal or tapering of doses at an individual level in any given study. The successful withdrawal was linked with low starting doses of antipsychotic medications, low scoring on the ABC, PRIMA, and Reiss rating scales, and co-existing epilepsy [66].

However, re-prescribing was necessary in some cases due to the re-emergence of symptoms [43,66] and unsuccessful withdrawal was associated with higher starting doses of psychotropic medications [2,66]. In Branford’s study, a plan to reduce/withdraw antipsychotic medication in 123 patients was achieved. The successful withdrawal was achieved in 43 patients, however, 42% (*n*** **=** **52) of cases attempting reduction or withdrawal resulted in re-prescribing/dose increase of antipsychotic medication [27]. One study suggests that following an initial relapse following an antipsychotic medication withdrawal attempt, further relapses are likely following future attempts [70].

The team effort was noted in one study as important for the successful reduction or withdrawal of psychotropic medications seen in the inpatient setting [71]. Similarly, A Cornwall-based study involving the development of a structured pathway to withdraw antipsychotic medications amongst adults with ID showed that withdrawal was achieved in 46.5% (33/71) of participants. A further 11.3% (8/71) achieved a dose reduction of over 50% [61]. The Cornwall-based study cited a concerted effort as necessary to achieve this level of success and required the involvement of all stakeholders from the outset, including the people with ID themselves and their carers. Another reported reason for success was the involvement of the multidisciplinary team throughout the entire withdrawal process and ongoing support for the patients and their carers following discontinuation [61].

Hesitancy from prescribers may also be a factor. As part of a discontinuation trial based in the Netherlands, ID physicians were unwilling to initiate discontinuations when there were concerns for restlessness, the presence of an autism spectrum disorder, previous unsuccessful attempts along with objections from legal representatives [29]. Conversely, a mixed-methods study showed that a large majority of support staff were willing to contribute to the achievement of antipsychotic medication discontinuation. That study noted also that the opinion of relatives, as a client’s legal representatives, weighed heavily in decisions on whether or not to reduce/discontinue antipsychotic medications [41].

Sheehan et al. investigated the feasibility of a structured web-based medication review tool for psychiatrists. The tool comprised measures of therapeutic benefits and adverse side effects. Seventy-nine people with ID were recruited and a total of 97 medication reviews were carried out over a 6-month study period and said it helped people with ID or their carers become more involved and promoted a collaborative decision-making process [62]. Another medication review too called “Systematic Tool to Reduce Inappropriate Prescribing” (STRIP) has also been found to be useful in identifying drug-related problems in people with ID. In a pilot study with 27 clients and their prescribers, a total of 127 drug-related problems were detected, mainly potentially inappropriate or unnecessary medications and after six months, 15.7% of the interventions were implemented [74].

## Discussion

This scoping review provides a systematic overview of studies exploring the prevalence of psychotropic medication use and evidence of effectiveness and adverse effects, as well as professional and lay practices around managing psychotropics. Patient outcomes associated with psychotropic medication use, such as reducing repetitive, stereotypic, and/or aggressive behaviours towards others, objects, or self were reported [33,46,49,52,53]. Side effects which include aggressive behaviour towards objects and/or self-injurious behaviour, reduced adaptive behaviour, and blunted affect were also described [2,41,50,54,55]. Healthcare providers felt there was a role for psychotropic medications in managing behaviours that challenge and also reducing or discontinuing psychotropic medications is sometimes warranted [41,74]. Access to multidisciplinary teams, guidelines, use of medication reviews, improved specialist training for healthcare providers, and an enhanced role for carers in decision-making were suggested to optimize appropriate psychotropic use.

Psychotropic medications, in particular, antipsychotic medications are commonly prescribed for people with ID to manage behaviours that challenge, often in the absence of a documented psychiatric diagnosis [32,37]. It is clear that there is a lack of evidence in respect of the question of the efficacy of psychotropic medications and studies are of questionable quality. Guidelines tend to be based on expert consensus and experience from practice. While providing a useful foundation for practice, the absence of strong evidence leaves them open to potential challenges. Undesirable side effects, such as weight gain and metabolic disturbance are both well-defined adverse effects of antipsychotic medications. The risk of inducing metabolic dysfunction varies among antipsychotics with a higher risk associated with new generation agents, such as clozapine and olanzapine which exhibit high affinity for 5-HT2C and Histamine H1 receptors [75,76]. Reliance on psychotropic medications for behavioural/emotional indications suggests a lack of sensitive methods available/accessible to diagnose and access to appropriate community supports for patients with ID.

Moreover, people with ID are at risk of receiving high doses of psychotropic medications and remain on them for long periods of time, often without review [77]. A practical solution to the issue of overprescribing psychotropic medications in this patient population would be to reduce and/or withdraw such medications however, evidence of successful withdrawal of medicines is lacking 4-74% [17]. Studies noted higher doses correlate with increased behavioural difficulties [2]. This is difficult to interpret as higher doses may be prescribed to manage behaviours but are also linked with side effects. This is concerning as not only are high doses associated with the paradoxical effect of more severe side effects, it also may hinder the tapering of medications in the future [2,66]. Avoiding destabilization of a patient during and following drug tapering is a concern for clinical staff and their family members [29,41]. Further information on the rate of relapse after psychotropic medication discontinuation in this cohort would be useful.

Collaborative practice in ID settings happens between psychiatrists, behavioural specialists, and professional and lay carers, to name some of the team. It is recognized as having positive impact on patient outcomes [41,61,71]. Given that ID is viewed as a specialist area and there is often a lack of a standardized structured model of care, research on the processes in services to support multidisciplinary communication and practice is often lacking. However, studies are beginning to explore this topic [78].

There is an emphasis on person-centred care and assessing the capacity of people with ID to manage their own medications and promoting independent living is receiving increasing attention [79,80]. Studies have called for training for guardians, prescribers, and disability and health professionals that addresses the intersection between physical and mental health and behavioural needs [29,40,44,59]. However, research is needed examining the effectiveness of education and training of clinical staff and patients, and families, as evidence regarding the effectiveness of such interventions to optimize prescribing is very mixed [81]. Furthermore, the issue of consent in relation to the medication regimens, assessment tools for supporting self-medication, and guidance for carers involved in the management and administration of medicines are not published/available. These guidelines and policies anecdotally exist at a local service level but assessment at an academic level or development of frameworks around the practices have not been carried out. Given the role of caregivers as legal guardians of people with ID and the time they spend ordering and administering medicines, further research should focus on understanding the burden and costs of this care and its impact on medicine management, which was beyond the scope of this paper.

Polypharmacy was also a notable feature across studies in this review [2,28,31,32,36]. Polypharmacy is attributable to co-morbidity, with behaviours that challenge, mental health and epilepsy being strong predictors of poypharmacy [57]. Furthermore, higher rates of excessive polypharmacy in people with ID compared to the community-dwelling general older population have been reported elsewhere [57,82]. The use of multiple medications and long-term use of medications in this population may cause preventable harm and more rigorous monitoring for adverse effects is required.

Study designs are frequently pragmatic, e.g. non-randomized study design or retrospective and this results in small sample sizes and heterogeneous cohorts, e.g. receiving different doses and different combinations of medications. A challenge with these studies is the unavailability of medical records or limited access to records and the variety of definitions of clinical outcomes recorded. Given that prescribing and deprescribing are dynamic and specific to the needs of individuals, analyzing medical records using defined daily doses, which is a calculation of the average maintenance dose per day for a drug [83], rather than individual doses may be useful. Large-scale retrospective analysis may be useful to inform rates of relapse. Indicators of medication appropriateness can be a judgement based or taken from explicit validated lists [84]. The use of explicit measurement tools may improve the ability to carry out these studies. Furthermore, capturing the true rate of benefit and adverse events experienced with changes to a drug, particularly during withdrawal is difficult. The selection of measures needs input from clinicians, support staff, and carers to help inform the most useful and feasible measures.

The qualitative studies were useful to explore the opinions of stakeholders not captured by retrospective cohort studies and trial design [29,41,60,61]. However, people with ID had few opportunities to become involved in studies about them [60]. As the evidence on relatives, caregivers, pharmacists, and nurses are non-existing or limited, research on these stakeholders could be useful in future research.

### Strengths and limitations of this review

The main strengths of this scoping review are that it provides a comprehensive overview of the available published literature on this topic, inclusive of a wide range of global-reaching databases. The review followed a rigorous methodological framework for scoping reviews, which assures consistency and structure of the search process and confidence in the reporting of findings. However, we did not assess the quality of the studies, as is typical for a scoping review. Furthermore, regarding patient and public involvement, there is an opportunity for engagement, potentially following published guidance on stakeholder involvement in systematic reviews [85]. Nonetheless, the findings and discussion points regarding gaps in research should help to define an agenda for future research on this topic.

## Conclusions

This study summarizes the published literature available, highlighting trends in psychotropic medication prescribing/use and patient outcomes in people with ID. These findings can inform prescribing interventions and highlight the need for timely and comprehensive patient outcome data, especially when the medications are used long-term use at high doses and what happens when these high doses are reduced or stopped.

## Supplementary Material

Supplemental Material

## Data Availability

The dataset used and analyzed during the current study are available from the corresponding author upon reasonable request.

## Abbreviations

UK: United Kingdom
ID: intellectual disability
JBI: Joanna Briggs Institute
PRISMA: Preferred Reporting Items for Systematic Reviews and Meta-Analyses
PCC: Population/Concept/Context
