# Supplementary material for: Management of psychotropic medications in adults with intellectual disability: a scoping review
Source: Ann Med. 2022 Sep 18;54(1):2486–99. doi: 10.1080/07853890.2022.2121853 (PMC9518601; doi:10.1080/07853890.2022.2121853)
Supplement: Supplemental Material [file IANN_A_2121853_SM0336.pdf]

Appendices

Supplementary Table 1: Preferred Reporting Items for Systematic reviews and Meta-Analyses extension for Scoping Reviews (PRISMA-ScR) Checklist

SECTION	ITEM	PRISMA-ScR CHECKLIST ITEM	REPORTED ON PAGE #
**TITLE**			
Title	1	Identify the report as a scoping review.	Title page 1,2
**ABSTRACT**			
Structured summary	2	Provide a structured summary that includes (as applicable): background, objectives, eligibility criteria, sources of evidence, charting methods, results, and conclusions that relate to the review questions and objectives.	2
**INTRODUCTION**			
Rationale	3	Describe the rationale for the review in the context of what is already known. Explain why the review questions/objectives lend themselves to a scoping review approach.	4, 5
Objectives	4	Provide an explicit statement of the questions and objectives being addressed with reference to their key elements (e.g., population or participants, concepts, and context) or other relevant key elements used to conceptualize the review questions and/or objectives.	5
**METHODS**			
Protocol and registration	5	Indicate whether a review protocol exists; state if and where it can be accessed (e.g., a Web address); and if available, provide registration information, including the registration number.	5
Eligibility criteria	6	Specify characteristics of the sources of evidence used as eligibility criteria (e.g., years considered, language, and publication status), and provide a rationale.	6
Information sources*	7	Describe all information sources in the search (e.g., databases with dates of coverage and contact with authors to identify additional sources), as well as the date the most recent search was executed.	5
Search	8	Present the full electronic search strategy for at least 1 database, including any limits used, such that it could be repeated.	5
Selection of sources of evidence†	9	State the process for selecting sources of evidence (i.e., screening and eligibility) included in the scoping review.	5
Data charting process‡	10	Describe the methods of charting data from the included sources of evidence (e.g., calibrated forms or forms that have been tested by the team before their use, and whether data charting was done independently or in duplicate) and any processes for obtaining and confirming data from investigators.	6
Data items	11	List and define all variables for which data were sought and any assumptions and simplifications made.	6
Critical appraisal of individual sources of evidence§	12	If done, provide a rationale for conducting a critical appraisal of included sources of evidence; describe the methods used and how this information was used in any data synthesis (if appropriate).	Not done

For Peer Review Only

SECTION	ITEM	PRISMA-ScR CHECKLIST ITEM	REPORTED ON PAGE #
Synthesis of results	13	Describe the methods of handling and summarizing the data that were charted.	6
**RESULTS**			
Selection of sources of evidence	14	Give numbers of sources of evidence screened, assessed for eligibility, and included in the review, with reasons for exclusions at each stage, ideally using a flow diagram.	7,8
Characteristics of sources of evidence	15	For each source of evidence, present characteristics for which data were charted and provide the citations.	8-15, Supplementary material
Critical appraisal within sources of evidence	16	If done, present data on critical appraisal of included sources of evidence (see item 12).	Not done
Results of individual sources of evidence	17	For each included source of evidence, present the relevant data that were charted that relate to the review questions and objectives.	8-15
Synthesis of results	18	Summarize and/or present the charting results as they relate to the review questions and objectives.	8-15
**DISCUSSION**			
Summary of evidence	19	Summarize the main results (including an overview of concepts, themes, and types of evidence available), link to the review questions and objectives, and consider the relevance to key groups.	15, 16
Limitations	20	Discuss the limitations of the scoping review process.	17
Conclusions	21	Provide a general interpretation of the results with respect to the review questions and objectives, as well as potential implications and/or next steps.	18
**FUNDING**			
Funding	22	Describe sources of funding for the included sources of evidence, as well as sources of funding for the scoping review. Describe the role of the funders of the scoping review.	18

JBI = Joanna Briggs Institute; PRISMA-ScR = Preferred Reporting Items for Systematic reviews and Meta-Analyses extension for Scoping Reviews.

* Where *sources of evidence* (see second footnote) are compiled from, such as bibliographic databases, social media platforms, and Web sites.

† A more inclusive/heterogeneous term used to account for the different types of evidence or data sources (e.g., quantitative and/or qualitative research, expert opinion, and policy documents) that may be eligible in a scoping review as opposed to only studies. This is not to be confused with *information sources* (see first footnote).

‡ The frameworks by Arksey and O'Malley (6) and Levac and colleagues (7) and the JBI guidance (4, 5) refer to the process of data extraction in a scoping review as data charting.

§ The process of systematically examining research evidence to assess its validity, results, and relevance before using it to inform a decision. This term is used for items 12 and 19 instead of "risk of bias" (which is more applicable to systematic reviews of interventions) to include and acknowledge the various sources of evidence that may be used in a scoping review (e.g., quantitative and/or qualitative research, expert opinion, and policy document).

From: Tricco AC, Lillie E, Zarin W, O'Brien KK, Colquhoun H, Levac D, et al. PRISMA Extension for Scoping Reviews (PRISMAScR): Checklist and Explanation. Ann Intern Med. 2018;169:467–473. doi: 10.7326/M18-0850

For Peer Review Only

Supplementary Table 2: Study Protocol and Search Strategy

The protocol is published here:
https://hrbopenresearch.org/articles/4-30

Examples of search strategies

Search OVID MEDLINE	Database:<br>Ovid MEDLINE(R) and Epub Ahead of Print, In-Process, In-Data-Review & Other Non-Indexed Citations, Daily and Versions(R) <1946 to May 12, 2021><br><br>1 Intellectual Disability/ 55,676<br>2 Developmental Disabilities/ 20,753<br>3 Cognitive Dysfunction/ 21,823<br>4 1 or 2 or 3 95,324<br>5 medication*.mp.370,422<br>6 medicine*.mp. 932,733<br>7 Serotonin Uptake Inhibitors/ 19,861<br>8 Monoamine Oxidase Inhibitors/ 10,234<br>9 Benzodiazepines/ 22,137<br>10 "mood stabiliser".mp. 128<br>11 Antidepressive Agents/ 44,765<br>12 Antipsychotic Agents/ 54,970<br>13 Psychotropic Drugs/ 21,420<br>14 Anti-Anxiety Agents/ 18,883<br>15 5 or 6 or 7 or 8 or 10 or 11 or 12 or 13 or 14 1,399,600<br>16 Medication Adherence/ or Guideline Adherence/ or "Treatment Adherence and Compliance"/ 54,619<br>17 Medication Therapy Management/ 2,334<br>18 discontinu*.mp. 133,913<br>19 withdraw*.mp. 139,784<br>20 16 or 17 or 18 or 19 320,252<br>21 4 and 15 4,132<br>22 20 and 21 273
Embase	#20. #13 AND #19 766<br>#19. #14 OR #15 OR #16 OR #17 OR #18 194,685<br>#18. 'medication therapy management'/de 12,294<br>#17. 'medication'/de AND 'cessation'/de<br>#16. 'medication'/de AND discontinuation 21,845<br>#15. 'patient compliance'/de 137,114<br>#14. 'medication compliance'/de 35,436

For Peer Review Only

	#13. #4 AND #12	15,521
	#12. #5 OR #6 OR #7 OR #8 OR #9 OR #10 OR #11	991,145
	#11. 'mood stabilizer'/de	8,403
	#10. 'benzodiazepine derivative'/de	42,846
	#9. 'anxiolytic agent'/de	21,467
	#8. 'psycholeptic agent'	15
	#7. 'neuroleptic agent'/de	81,424
	#6. 'antidepressant agent'/de	102,380
	#5. 'drug therapy'/de	802,478
	#4. #1 OR #2 OR #3	240,327
	#3. 'developmental disorder'/de	36,910
	#2. 'cognitive defect'/de	178,749
	#1. 'intellectual impairment'/de	31,294

For Peer Review Only

Supplementary Table 3: Data extraction

Title, Authors, Year & Country of Publication	Aims of Study	Methodology	Intervention	Outcomes	Key Findings	Limitations
Ahmed Z, Fraser W, Kerr MP, Kiernan C, Emerson E, Robertson J, Felce D, Allen D, Baxter H, Thomas J. Reducing antipsychotic medication in people with a learning disability, 2000, UK	To investigate the feasibility of antipsychotic drug reduction in people with learning disability and the factors influencing the outcome	**Study Design** – Randomised controlled trial **Population** – people with ID and were 18 or older **Sample –** 56 participants **Study Setting** – people living in institutions or community residential home	Each participant was monitored?) for 6 months with baseline assessment, followed by 4, monthly drug reductions of 25% of the baseline chlorpromazine equivalent dose	ABS, ABC, DISCUS questionnaires were administered, eight and medication regimens were recorded, behaviours was directly observed for randomly selected 1.5hr periods during the 2nd, 3rd and 4th weeks of the month.	Twelve participants (33%) completed full withdrawal and had the absence of increase in maladaptive behaviour	**Funding:** Nil **Conflict of Interest**: Nil declared. **Acknowledgements:** clinical staff listed **Limitations:** small numbers in study, time scale for drug reduction may have been too rapid, study was not double blinded
Aman, M. G., & Singh N. A Critical appraisal of recent drug research In mental retardation: The coldwater studies. 1986, New Zealand.	To assess and evaluate public and professional attitudes towards pharmacological treatment in people with ID, by describing recent research on the use of psychotropic medications within this cohort.	**Study Design** - Critical appraisal, review of studies that have demonstrated minimal or no adverse effects associated with antipsychotic medication use in individuals with ID. **Population** – N/A **Sample –** N/A **Study Setting** – N/A	N/a	N/a	**Studies Suggest Adverse Effects -** Findings suggest that use of antipsychotics may interfere with adaptive behaviour and learning of institutionalised individuals with ID. **Research that Provides Conflicting Results with Those of the Coldwater Studies -** A number of studies demonstrated that use of antipsychotics suppresses stereotypic behaviour in ID patients.	**Funding** – Nil declared. **Conflict of Interest** – Nil declared. **Acknowledgements** – Nil declared. **Limitations -** Subject selection in the studies that suggest adverse effects. Subjects were commonly selected for the Coldwater Participants were recruited due to a lack of benefit from pharmacotherapy. and thus aimed to prove that pharmacotherapy had no place in the treatment of

For Peer Review Only

						behavioural issues in patients with ID.
Bowring, D. L., Totsika, V., Hastings, R. P., Toogood, S. and McMahon, M. Prevalence of psychotropic medication use and association with challenging behaviour in adults with an intellectual disability. A total population study. 2017, UK/Jersey; Channel Islands	To-examine limitations in existing studies by investigating the prevalence of psychotropic medication use in the total population of adults with ID in Jersey, Channel Islands in receipt of or previously linked with support services. This study aims to examine the association of psychotropic medication use with behaviours that challenge / types of behaviours that challenge. The paper explored the association between prescribing patterns and behaviours that challenge.	Study Design - A total population sampling method was used. **Population** – Adults (>18) with ID. **Sample**: 265 adults with ID who were receiving or had received support from services in Jersey. **Study Setting** - Participants were identified from multiple sources including the Health and Social Services administrative database and Functional Analysis of Care Environments (FACE) database.	Researchers completed two surveys for each participant via face-to-face meetings with a proxy informant. Informants were either family members or key workers within a supporting organisation. All data were collected over a period of 12 months (2013 –2014). -	1. Medication data<br><br>2. Behaviours that challenge data<br>-	The majority of participants lived in either congregate care (40.8%) or with family (34.3%). 26.4% of participants had a psychiatric condition.<br><br>Psychotropic medications were used by 37.73% of participants with antipsychotics being the most common at 21.89%. Most commonly used were atypical antipsychotics used by 15.09%. Common medications included Risperidone (n=16) and Olanzapine (n=13). Antipsychotics were used by 7.92% of participants. The second largest group of psychotropic medications was antidepressants at 17.38%, the majority of these were SSRIs used by 12.83%.<br><br>Almost one-third of participants were prescribed psychotropics above the defined daily dose (DDD): 30.43% (n=42). GLMs indicated a statistically significant association between	**Funding** - Nil declared. **Conflict of Interest** – Nil declared. **Acknowledgements:** The authors are thankful to all participants would like to thank all service users, their families, service providers and support staff who contributed to this research. We are very grateful to the following In addition to the research staff who contributed to data collection. In addition to Jessica Ramos De Castro, Ashley Kaye, Isabel Lewis, Michael Sleath, Doc Snook and Carol-Ann Doherty. We are also very grateful to the States of Jersey Health and Social Services Department for their support for this research. **Limitations** - Findings apply only to the ID population in Jersey who are linked with support services; there may be adults with ID not known to services that were not included.<br><br>The sample size was also relatively small compared

					psychotropic medication and the presence of a psychiatric diagnosis, behaviours that challenge, older age, type of residence and gender. Male gender was associated antipsychotic medication use. Behaviours that challenge was a predictor of medication use after controlling for other variables. The data indicates that there may be differences in prescribing patterns associated with different types of behaviours that challenge.	with other studies of that time.<br><br>The reliance on proxy informants to report medication use for those living in family or independent settings (133 participants).
Bradley, E. and Cheetham, T. The use of psychotropic medication for the management of problem behaviours in adults with intellectual disabilities living in Canada. 2010, Canada.	To provide a Canadian perspective on the use of psychotropic medication in the management of problem behaviours in adults with ID in Canada in the context of Canadian health and social services, clinical practices, medical training and factors that have shaped these over the past few decades.	**Study Design-**<br>1. Exploratory/ descriptive study<br>2. A Survey questionnaire was e-mailed to the membership of the Canadian National Coalition in Dual Diagnosis and colleagues in the clinical networks associated with the Primary Care Guidelines Initiative.	1.Scoping review of associated available literature<br><br>2. A Canadian wide survey of psychotropic medication management and ID.<br><br>3. Comparison by the authors of circumstances that affect health care and the use of psychotropic medications for people with ID in Canada versus the UK, i.e. training/ policy/ legislation etc.	A total of 57 survey responses were recorded. 55% of informants (from 6 provinces) were recruited through the Canadian National Coalition. 45% (N = 25) of informants were recruited through informal ID clinical networks to which the authors belong. These 25 individuals, were estimated by the authors to include around 80–90% of Canadian physicians whose primary practice is in ID.	In Canada there are no national/ provincial/ territorial policies or guidelines on the use of psychotropic medication for the management of problem behaviours in ID, nor any national policy to address the specific needs of people with ID. Draft guidelines exist in some provinces. There are no requirements for physicians prescribing these medications to have training in the care of people with ID.	**Funding** - Nil declared.<br><br>**Conflict of Interest** - Nil declared.<br><br>**Acknowledgements** - The authors much appreciate the scholarly assistance provided by Marika Korossy.<br><br>**Limitations** -<br>It is not known whether the questionnaire was anonymous and this may have affected the findings as the authors mention that some of the informants were

review only

					Services appear to be more crisis-reactive versus proactive/ preventative in the UK, with no centralised approach.<br><br>Accreditation standards in Canada, apply only to organisations and not to individual prescribers outside these organisations. Standards for medication management refer to health settings that have in-house pharmacies and adjustments would be required for these standards to apply to community pharmacies.<br><br>Psychiatrists in Canada tend to follow American Psychiatric Association and Canadian Psychiatric Association practices. Most intellectual disabilities community agencies do not have access to experienced ID nurses to assist in medication-related issues. Family physician training has no specific requirements for exposure to patients with ID/ no training in ID-specific medication related issues. Referral	colleague via professional networks.<br><br>Small sample size.<br><br>This study is > 10 years old, guidelines may have changed since then.

For Peer Review Only

					to specialist dual diagnosis psychiatric services is difficult due to the paucity of such services; the family physician manages the patient as best he/ she can with limited training and experience.	
Branford D.<br>A Review of Antipsychotic Drugs Prescribed for People with Learning Disabilities Who Live in Leicestershire, , 1996, England (UK).	To assess the continuing need for antipsychotic drug therapy in individuals with learning disabilities for whom they were prescribed.	**Study Design** – A five stage process: recruitment, case review, programme for change of drug therapy, drug change and follow-up.<br>**Population** - All patients under NHS care, the antipsychotic drug review programme.<br>**Sample** - 198 patients prescribed antipsychotic drugs were reviewed (58% of the identified population).<br>**Study Setting** - Various forms of NHS residential accommodation.	Review of past prescribing and notes with reference to previous changes to drug therapy. Completion of Aberrant Behaviour Checklist, Reiss and PIMRA. A case review focused on withdraw of antipsychotics where; there was no clear benefit from the prescribed antipsychotic, significant time (>5 years) had elapsed since the original prescribing, the degree of benefit achieved was at the expense of a reduced level of mental or physical functioning or alternative methods of containing the behaviours that challenge were available.	Out of the 198 patients reviewed, 123 underwent a reduction of antipsychotic drug dose.<br><br>Reduction occurred where patients were not receiving Lithium in addition to an antipsychotic drug, receiving low doses (less than 100 mg equivalent to chlorpromazine) of antipsychotic drug and those who achieved low scores on the Reiss screen sub-scales of Aggression, Psychosis, Paranoia, Dependent Personality, over activity and Self-Injury, and lower scores on the ABC subscales of Irritability and Hyperactivity.	Twelve months after the initial dose reduction, 31 (25%) patients remained free of antipsychotic drugs. The remaining patients had different outcomes. For 12 patients, although complete withdrawal of antipsychotic drug therapy had been achieved, re - prescribing was necessary following a deterioration of behaviour. Forty patients suffered a deterioration in behaviour following some reduction of dose, and there was no clear outcome for the remaining.	**Funding** – Nil declared.<br>**Conflict of Interest** – Nil declared.<br>**Acknowledgements** – Were stated to the following individuals: Dr R. A.Collacott and Dr D. A.Hutchins who supervised the study; Drs Bhaumik, Ismail, Hauck, Agrawal and Tanna for their assistance with the review of patients; the nursing and care staff who completed assessments of patients; David Arrowsmith, for supporting the transfer to the Glenfrith Division; Margaret Black for her assistance with the drug history programme; to Liliane McManus for typing the manuscript.
Branford, D.<br>Factors associated with the successful or unsuccessful	This study aims to analyse data related to outcomes of people with intellectual disability in attempt to identify factors associated with	**Study Design** - A preliminary survey identified people with ID living in Leicestershire who routinely receive antipsychotic drugs.	Reduction/ Withdrawal of antipsychotics.<br><br>Doses were reduced on a monthly basis unless an	Patient outcomes related to drug reduction/withdrawal.	In 52 (42%) of the 123 patients, dose reduction or withdrawal led to a worsening of behaviour which in turn resulted in re-prescribing or a dose	**Funding** - Nil declared.<br>**Conflict of Interest** - Nil declared.<br>**Acknowledgements** - Many Thanks to Dr R. A. Collacott and Dr D. A.

withdrawal of antipsychotic drug therapy prescribed for people with learning disabilities. 1996, United Kingdom.	successful withdrawal of antipsychotic drugs.	Between 1991 and 1993 a systematic review was carried out on 198 of these.<br>**Population –** People with ID who receive antipsychotic drugs**.**<br>**Sample –** Information from medical notes was available for 198 patients. Of the 198 patients, reduction and withdrawal of drug therapy was planned in 123 with planned regular reviews.<br>**Study Setting –** NHS accommodation and non-NHS accommodation.	increase in severity or frequency of behaviours that challenge occurred.		increase to counteract this. In 31 (25%) cases the medications were successfully withdrawn.<br><br>Of the patients who successfully withdrew from antipsychotics, some characteristics were noted in this study; they were mostly on low doses of antipsychotics (less than 100mg daily equivalent to chlorpromazine), they scored low on the ABC, PRIMA and Reiss rating scales and often had a co-existing epilepsy.<br><br>In contrast, in those in which drug withdrawal was unsuccessful, doses tended to be higher (greater than 100mg daily equivalent to chlorpromazine). In addition, following completing of the rating scales suggested that those with aggressive behaviours and those with high scores on the ABC sub scale of irritability, stereotypy and hyperactivity are less likely to be successful.	Hutchins, under whose supervision this work was carried out, to Cathy Thorp of the Leicester Information Co-ordination Centre, and to Liliane McManus for typing the manuscript.

For Peer Review Only

Deb, S., Unwin, G. and Deb, T. Characteristics and the trajectory of psychotropic medication use in general and antipsychotics in particular among adults with an intellectual disability who exhibit aggressive behaviour. 2015, UK.	To examine the trajectory of psychotropic medication use in the population with ID and their relationship with different demographic and aggression related variables using a prospective study design.<br><br>.	**Study Design** - Prospective study design.<br>**Population** – Adults with ID and aggressive behaviour.<br>**Sample** – 100 adults with ID and aggressive behaviour identified through review of case notes of all patients attending each outpatient clinic within the last 12 months.<br>**Study Setting –** Community, Psychiatric clinic based.	One author (GU) collected information on various assessment scales by interviewing either a paid or non-paid family carer who knew the patient well. The following assessments were done:<br><br>1. Aggressive behaviour was measured using the Modified Overt Aggression Scale (MOAS). Broader Problem behaviours were measured using the Aberrant Behaviour Checklist Community Irritability (ABC-I) and other sub-scales (ABC-Lethargy, ABC-Stereotypic behaviour, ABC-Hyperactivity, and ABC-Inappropriate speech)<br><br>2. Psychiatric disorders were assessed using the Mini Psychiatric Assessment Scale for Adults with Developmental Disabilities (Mini PAS-ADD) interview<br><br>3. Data was collected on the psychotropic medications prescribed	1. Presence of aggressive behaviour<br><br>2. Presence of psychiatric disorders<br><br>3. Data on psychotropic medications (regular and PRN) prescribed to each patient.<br><br>4. Demographic data: age, gender, severity of ID, physical disorders, place of residence etc.	Psychotropic medications were used for 89% of patients at baseline (T1) and 90% at 6months' (T2) follow-up.<br><br>Risperidone was the most commonly used antipsychotic medication. Other commonly used medications were SSRIs and mood stabilisers, carbamazepine and sodium valproate and in a high proportion of cases these drugs were used to treat epilepsy. 45% received more than one (polypharmacy) psychotropic medication at T1; however, this proportion decreased to 41% at T2. A similar proportion received antipsychotics at T1 (75%) and T2 (73%), with polypharmacy of antipsychotics remaining similar at T1 (10%) and at T2 (9%).<br><br>23% and 20% of patients received over 300mg/day of chlorpromazine equivalent dose of antipsychotics at T1 and T2 respectively. However, there was an overall significant reduction in the severity	**Funding-** The study was funded by the Baily Thomas Foundation, UK.<br>**Conflict of interest -** Nil declared.<br>**Acknowledgments -** The study was funded by the Baily Thomas Foundation, UK.<br>**Limitations -** Generalisability: Data was collected from 10 clinics covering 6 diverse Trusts in rural and urban areas and varying social economic classifications. Despite this, generalisability may not be possible as the data may represent more local practices/ a nationwide survey was not undertaken / the no. of patients recruited was relatively small.<br><br>The relatively high rate of prescribing relates to a psychiatric clinic sample of adults specifically with aggressive behaviour, not a population-based sample. Therefore, they are not representative of prescribing rates among people with ID more broadly.

			to each patient at each time point.		of aggressive behaviour between T1 and T2. Higher doses of antipsychotic prescribing were positively correlated with more severe aggressive behaviour, physical aggression towards objects, self-injurious behaviour and increasing age. There was no significant association with other demographic variables, physical health conditions or psychiatric diagnosis. Neither was there any significant correlation between mean aggression severity score change and antipsychotic daily dose change between T1 and T2.	
de Kuijper, Mulder, Evenhuis, Visser, and Hoekstra. Effects of controlled discontinuatio n of long-term used antipsychotics on weight and metabolic parameters in individuals With	To evaluate the effects of discontinuation of long - term antipsychotics on various health parameters which include waist circumference, weight, body mass index (BMI) and on parameters of the metabolic syndrome.<br><br>A secondary aim to this study was to Investigate a possible link between genetic polymorphisms and medication factors	**Study Design** – A controlled, discontinuation study of long-term antipsychotics prescribed for behavioural disturbances. **Population** – Residents with intellectual disabilities from three care settings in the Netherlands. **Sample** – 99 residents which were recruited from each of the three residential care settings.	Discontinuation of long-term antipsychotic medication in 14 or 28 weeks. Each group involved eight intended gradual dose reductions of approximately 12.5% of the initial dose that was done every 2 weeks in the 14 week group and every 4 weeks in the 28 week group.	Fasted glucose levels, triglycerides, high-density lipoproteins, low-density lipoproteins and total blood cholesterol. Height, weight, waist circumference and blood pressure were also measured.	43 participants had achieved complete discontinuation. Of these, 7 were reinstated on antipsychotics and 36 were completely off them at follow-up. In those that had not achieved full discontinuation the mean maximum dose reductions were 41% and 48% in the 14 and 28 week groups respectively. Results showed that	**Funding -** Nil declared. **Conflict of Interest -** Nil declared **Acknowledgements -** Nil declared. **Limitations -** The lack of measurement of weight, waist circumference, blood pressure and fasting lipids and glucose before treatment with antipsychotics started. -Relatively small sample size / no control group

Intellectual Disability. 2013, The Netherlands.	and the metabolic outcomes of discontinuation.	**Study Setting** – The study took place in residential settings of three care providing organisations.			discontinuation of antipsychotics led to a significant decrease in waist circumference, weight, BMI, and systolic blood pressure. There was no significant difference between results of the 14 week group and the 28 week group.<br>-In those that did not achieve full cessation of antipsychotics, reduction in weight and BMI was seen even after small dose decreased.<br>-A higher baseline dose of antipsychotic was associated with a larger decrease in waist circumference, weight and BMI in participants that achieved full discontinuation of antipsychotics.	No application of Bonferroni corrections which may have yielded false positive results.
De Kuijper, G. M. & Hoekstra, P. J. Physicians' reasons not to discontinue long -term used off-label antipsychotic drugs in people with intellectual disability.	To investigate the influence of participant and setting related factors on physician decision not to discontinue off - label prescribed antipsychotics.	**Study Design -** cohort study, nested in a discontinuation trial.<br>**Population -** People with ID and their support professionals, nurses, behavioural scientists and physicians.<br>**Sample -**3299 clients and their health professionals.<br>**Study Setting -** Living facilities of six service	N/a	**1.)** Prevalence of antipsychotic drug use in the study population<br>**2.)** Duration of use<br>**3.)** Reasons of physicians not to discontinue the prescription of antipsychotics in those participants who used off-label antipsychotics for more than a year	Of the 3299 clients, 977 used one or more antipsychotic drugs. The prevalence of antipsychotic drug use was 30%. Reasons for use were:<br>• 5% of cases, a chronic psychotic disorder classified according to Diagnostic System Mental Disorders,	**Funding** - This study was funded by Stichting Zorgondersteuningsfonds , Soesterberg and the Netherlands.<br>**Conflicts of Interest –** Nil declared.<br>**Acknowledgments –** Nil declared.

For Peer Review O

2017, Netherlands		providers for people with ID spread throughout the Netherlands.			Fourth Edition, criteria.<br>• 25%, present or past (suspected) non-schizophrenia-related psychotic symptoms.<br>• 69%, behaviours that challenge.<br>-Physicians were willing to discontinue their prescriptions in 51% of cases, varying from 22% to 87% per service provider. The likelihood of this varied from 0.19 to 13.95 per prescriber. The variable living situation with care and support' and 'behaviours that challenge 'were associated with a higher chance of discontinuation.<br>- The reasons for not discontinuing off – label antipsychotics were concerns for symptoms of restlessness, the presence of an autism spectrum disorder, previously unsuccessful attempts to discontinue and objections against discontinuation of legal representatives.	
De Leon, J.,Greenlee, B., Barber, J., Sabaawi, M. & Singh, N.	To establish a framework for the use of new generation antipsychotic (NGA) drugs (aripiprazole,	**Study Design -** Narrative review<br>**Population -** adult individuals	New generation antipsychotic (NGA) drugs in adult individuals with ID.	Expert consensus guidance and well-controlled comparison trials, a set of guidelines are presented to inform	**1.)** NGA drugs are less toxic than clozapine, which may have a role in more difficult cases and may have some	**Funding /Conflict of Interest** Dr.de Leon-declared<br>**Limitations -** The application of

Practical guidelines for the use of new generation antipsychotic drugs (except clozapine) in ddult individuals with intellectual disabilities. 2009, USA.	olanzapine, paliperidone, quetiapine, risperidone, and ziprasidone) in adult individuals with intellectual disabilities by utilizing the prescribing information and reviewing the available literature on the relevant neuropsychiatric Disorders.	with intellectual disabilities **Sample -** N/a **Study Setting-** USA.		initiation, dosing and monitoring of use in adults. In these guidelines practical information is provided on drug–drug interactions and adverse drug reactions, and a brief review of discontinuation syndromes, potential for abuse, use during pregnancy and cost considerations.	advantages over conventional antipsychotics. **2.)** They produce less EPS, but they are not completely free of them. **3.)** Metabolic syndrome complications may be worse with some of these NGAs versus high-potency conventional antipsychotics. **4.)** The NGA drugs are definitely more expensive than the conventional antipsychotics.	this information in a particular situation remains the professional responsibility of the practitioner. The procedures contained in these guidelines may not fully account for all of the possible risks of treatment in this population because of the limited studies available.
Drmici S and Franic T. Effect of olanzapine on disruptive behaviour in institutionalized patients with severe intellectual disability - a case series. 2008, Croatia.	To expand existing knowledge regarding the efficacy of atypical antipsychotics for the treatment of behaviours that challenges in people with ID. This study focused on olanzapine.	**Study Design** - This study is a partially prospective, open label case series report. **Population -** Institutionalised patients with severe intellectual disability. **Sample -** 33 participants who were institutionalised due to the presence of ID, behaviours that challenge and the lack of appropriate family support. **Study Setting –** Institution for adults with ID.	Introduction of olanzapine following a drug free period in those previously treated with thioridazine.	Outcome of olanzapine treatment was measured using the global improvement and efficacy index measures of the clinical global impression-global improvement (CGI-GI) scale.	All participants were previously treated with thioridazine for disruptive behaviour. The duration of such treatment ranged between 8-36 months with the mean being 18 months. During the drug free period 21 (63.6%) participants demonstrated one or more disruptive behaviours in which olanzapine was then commenced. The caregiver rated five point scale scores declined significantly between day 0 and 30 and between day 30 and	**Funding -** Nil declared. **Conflict of Interest -** Nil declared. **Acknowledgements-** Nil declared. **Limitations -** No placebo. No blinding. 5 point scale used by carer givers is subjective and open for misinterpretation.

For Peer Review

					day 60. Each of the seven observed disruptive behaviours declined between days 0 and 30 ($p<0.05$). Two types of the behaviours showed further decline until day 60; self-injurious behaviour and attacks to objects/damage to property.	
Eady, N., Courtenay. & Strydom, A. Pharmacological Management of Behavioural and Psychiatric Symptoms in Older Adults with Intellectual Disability. 2014, UK.	To explore the pharmacological treatment of behavioural / psychiatric symptoms and disorders in older adults with ID.	**Study Design** - Review **Population –** N/A **Sample** - N/A **Study Setting –** N/A	N/a	**1.)** Mental Health Disorders in Older Adults with ID. **2.)** Medication Management of Problem Behaviour in Older Adults with ID. **3.)** Polypharmacy and Prescribing Patterns in Older Adults with ID.	The growing population of older adults with ID has an increased risk for aging - related health issues such as dementia, on a background of lifelong disabilities and physical / psychiatric co-morbidities. Medication management of behavioural and psychiatric problems may be complicated by a higher risk of adverse events, lack of decision making capacity, and complex care networks. -Medication should generally be started at low doses, with careful titration against response and regular monitoring for side effects.	**Funding** - This work was partially funded by a Wellcome Trust Strategic Award conferred upon The London Down Syndrome (LonDownS) Consortium. **Conflicts of Interest** - Dr. Strydom - declared Dr. Courtenay and Dr. Eady do not have conflicts **Limitations –** N/a
Erickson, S. R. In - Home comprehensive medication	To develop and pilot a workflow for an in - home Comprehensive Medication Review	**Study Design** - pilot feasibility study.	CMR is a model in which pharmacists conduct in-depth interviews and assessments of patients’	**1.)** Patients’ characteristics included demographics, documented disease	The mean number of medications taken per person, including scheduled and as needed	**Funding –** This work was supported by a grant from Community Living

Peer Review Only

reviews for adults with intellectual or developmental disability: A pilot study. 2020, USA.	(CMR) program for adult patients who have an Intellectual or Developmental Disability (IDD).	**Population** - The subjects were patients who have an IDD, aged 21 years or older. Subjects were clients of CLS, a support agency and had 5 or more prescription medications documented for the treatment of chronic physical and mental health diseases / disorders.<br>**Sample** - 15 patients.<br>**Study Setting -** 12 group homes located throughout Wayne County, MI, USA.	medication regimens. Once problems are identified, interactions occur between the pharmacist and prescriber, among the pharmacist and patient and caregiver, and between the pharmacist and other pharmacists.	states, medication categories, and IDD - related diagnosis.<br><br>**2.)** A description of the type and frequency of occurrence of each Medication Related Problem (MRP) category and the overall average number of MRPs per patient was calculated.<br><br>**3.)** Patient-specific unique routes of medication administration other than by mouth were documented.<br><br>**4.)** Detailed descriptions of the interventions recommended by the pharmacist were provided along with the acceptance rate of recommendations made to prescribers.<br><br>**5.)** The amount of time taken to complete the primary steps of the project was summarized	was 12.1 ±4.0 (range 6 - 20). 13 of the 15 patients were taking at least 1 psychotropic medication. Of those taking psychotropics, 5 were taking 2 medications, another 5 were taking 3, 2 were taking 4, and 1 was taking 5. The most common medication categories prescribed were antipsychotics (23 medications), seizure medications (21), and laxatives (18). Patients were found to have an average of 2.4 ±1.5 MRPs. All real-time recommendations made directly to prescribers by the pharmacist were accepted and implemented.<br><br>6 patients had to have their medications crushed and mixed with apple sauce, yogurt, or other thickener to enhance their ability to swallow the medication. 2 patients required assistance with drug-delivery devices, including eye drops and an inhaler. The average amount of time preparing, conducting, and	Services (CLS; 17-PAF05960), Wayne, MI.<br>**Conflict of Interest –** The author declares no relevant conflicts of interest or financial relationships.<br>**Limitations –**<br>This study was conducted using the population of a single support provider agency. Such agencies vary in their medication management policies / procedures, which could potentially lead to differences in prevalence and types of MRPs recorded.<br>-Small sample size.<br>-Patients in this study primarily had Medicaid insurance, whose prescription drug policies vary from state to state, which could introduce differences in the types of MRPs.<br>-This study did not examine the clinical outcomes associated with identification and remediation of MRPs, nor did it examine the cost and utilization outcomes.

					following up on each patient's CMR was 19.3 ±9.6 minutes to prepare, 39.0 ±14.0 minutes to conduct the in-home data collection and assessment, and 26.7 ±20.7 minutes to follow-up, which included consulting drug information material, writing recommendations, and contacting prescribers. The longest overall CMR session was 170 minutes.	
Espadas, C., Ballester, P, Londoño, A. C., Almenara, S., Aguilar, V., Belda, C., Pérez, E. & Peiró, A.M., Multi-morbidity and Psychotropic Polypharmacy Among Participants with Autism Spectrum Disorder with Intellectual Disability, 2020, Spain.	The study aimed to evaluate medication - related safety, drug - drug interactions, and psychotropics prescription trends in adults with Autism Spectrum Disorder (ASD) and ID.	**Study Design** - Observational and multi-centric pharmacovigilance study between January 2015 and January 2018 (36 months). **Population** - Subjects with ASD and ID. **Sample** – 83. **Study Setting** – 4 different residential facilities, linked to the Alicante General Hospital health network, Spain.	N/A	**1.)** Drug prescription recorded as well as the presence of polypharmacy, defined as: **(a)** The use of several drugs at once (≥ 4), or **(b)** The prescription of higher doses than the recommended daily dose. **2.)** Multi-morbidity (defined as the presence of ≥ 4 chronic health conditions).	A mean of 4 medicines per subject (IQR, 3–6) was found. This represents a prevalence of 57% of polypharmacy. 13% of the participants had doses outside the range indicated on the technical sheet. 48% were taking antipsychotics, 22% anticonvulsants, 17% anxiolytics and 13% antidepressants. The most prescribed group of drugs were antipsychotics. Risperidone was the most used (20% of total antipsychotics prescriptions), at a High Recommended Daily Dose (HIGHRDD)	**Funding** – The project was funded by the Alicia Koplowitz grant for research in neurosciences (2014 – 2016) and the FISABIO (Foundation for the Promotion of Health and Biomedical Research of Valencia Region) research grant for residents in its Alicante centre (2015 –2018). The author P. Ballester also received a predoctoral grant from Fundación La Caixa (grant PT13/0001). **Conflict of Interest** – Nil declared. **Acknowledgements** - The authors thanked study participants, their families and staff from the Spanish autism

					together with Olanzapine and Quetiapine.<br><br>Topiramate was the most prescribed anti-convulsant drug. Valproic acid was prescribed at HIGHRDD in 22% of instances where prescribed. The most common anxiolytics prescribed were Clonazepam, Clorazepate and Diazepam.<br><br>Most increased health risks were related to CNS depressant, anti-dopaminergic (extrapyramidal symptoms and neuroleptic malignant syndrome) or cardiologic adverse effects (e.g. QTc -prolongation). There was no routine clinical protocol to monitor or mitigate these adverse effects.	associations (SanRafael, EDUCATEA, APNAV, and Infanta Leonor) for their involvement.
Haessler, F. *et al.*, Zuclopenthixol in Adults with Intellectual Disabilities & Aggressive Behaviours: Discontinuation study, 2007, Germany.	To investigate the effects of Zuclopenthixol on aggressive behaviour in patients with ID by randomly withdrawing it after a 6 -week period of open treatment.	**Design** - Randomised, double – blind, placebo - controlled withdrawal study with parallel groups.<br>**Population** - people aged 18 - 50 years, with mild to moderate ID.<br>**Sample** – 49, with 39 taking part in the randomised trial;	49 individuals with mild to moderate ID received open treatment with Zuclopenthixol for 6 weeks for exacerbations of aggressive behaviour. Behavioural problems were rated on the Disability Assessment Schedule (DAS). Zuclopenthixol was	The number of adverse events and possible symptoms of withdrawal, (such as nausea, insomnia, and diarrhoea), were recorded and did not differ between the groups.	The placebo subgroup showed more aggressive behaviour than the continuing subgroup, as indicated by outcomes observed by external rates on the MOAS.<br>The results indicate that discontinuation of Zuclopenthixol in this population leads to an	**Funding** – Nil declared.<br>**Conflict of Interest** – Nil declared.<br>**Acknowledgements** - The study medication and placebos were provided by Bayer Vital GmbH, D 162,51368 Leverkusen, Germany.

For Peer Review Only

		'responders' (20 in the placebo group, 19 in the Zuclopenthixol group).<br>**Study Setting** – 6 German intellectual disability centres.	administered at a dosage of 2 -20 mg per day. The Modified Overt Aggression Scale (MOAS) was administered every 2 weeks. Routine laboratory tests of prolactin and serum Zuclopenthixol were conducted.		increase in aggressive behaviour.	
Haessler, F. *et al.* 2008. A Double - Blind Placebo - Controlled Discontinuatio n Study of Zuclopenthixol for the Treatment of Aggressive Disruptive Behaviours in Adults with Mental Retardation – Secondary Parameter Analyses, Germany.	To evaluate the effects of Zuclopenthixol withdrawal.	**Study Design -** A multicentre, randomized trial, double - blind, placebo - controlled parallel group comparison with an enrichment design.<br>**Population -** All patients between the age of 18 and 50 years, diagnosed with intellectual disability displaying aggressive behaviour for at least 14 days per month within the past 6 months. Scored <39 for the total score of the Disability Assessment Schedule (DAS) Complex VII.<br>**Sample -** 39, 19 received Zuclopenthixol and 20 received placebo.<br>**Study setting -** Intellectual disability centres in Germany.	Six Week open label phase where Zuclopenthixol was administered at a dose of 2 to 20 mg per day. Routine laboratory tests and measurements of prolactin and serum levels of Zuclopenthixol were conducted. Weekly safety assessments included measures of extrapyramidal signs, vital signs and weight. Disability Assessment Schedule (DAS), improvement on the Clinical Global Impression Scale (CGI-I), and the Nurse's Observation Scale for Inpatient Evaluation (NOSIE), Modified Overt Aggression Scale (MOAS) measures used.	The Zuclopenthixol group had a significantly greater decrease in the weighted MOAS total score than did the placebo group.<br>In the placebo an increase in behaviours that challenge after randomization occurred. Patients receiving placebo also had more problems with disruptive behaviour when ratings from the staff members were used.	10 patients (20%) discontinued the study due to insufficient therapeutic effect or adverse events in the open period. Zuclopenthixol was significantly superior to placebo for maintaining a low level of aggressive behaviour.	**Funding** – Nil declared.<br>**Conflict of Interest** – Nil declared.<br>**Acknowledgements** - This study was supported by the Bayer Vital GmbH, D 162, 51368 Leverkusen, Germany.
Haessler, F. *et al.*, Effects of Zuclopenthixol on Aggressive Disruptive	To investigate long - term effects of Zuclopenthixol; all patients of this study receiving Zuclopenthixol after trial were examined	**Study Design –** A multi-centre, open-label, prospective follow-up on a randomized, placebo-controlled withdrawal	Zuclopenthixol was administered at a dose of 2 – 20 mg per day adjusted once or twice daily when judged to be	Patients who received Zuclopenthixol showed significant benefits for all 3 dependent efficacy measures.	Patients still treated with Zuclopenthixol after 2 years (n = 21) benefitted, compared to the dropouts (n = 10).	**Funding** – Nil declared.<br><br>**Conflict of Interest** - Disclosures of interest for Prof. Haessler: declared.

For Peer Review Only

Behaviour in Adults with Mental Retardation – a 2 - year follow - up on a withdrawal study, 2011, Germany.	for efficacy and safety at a reporting date 2 years later.	trial where a short-time withdrawal trial of 12 weeks was extended at open label to 2 years.<br>**Population -** All patients were diagnosed as having intellectual disability.<br>**Sample -** 49 with 39 individuals receiving either Zuclopenthixol (n=19) or placebo (n=20).<br>**Study Setting –** 10 intellectual disability centres in Germany.	necessary by the clinician.<br>To estimate rates of disruptive behaviour the weighted score of the modified overt aggression scale (MOAS), the score complex VII of the disability assessment schedule (DAS) and the clinical global impression scale (CGI-1) were used.	For the DAS, an improvement of 3 points was measured however, this did not reach the cut - off for non - problematic behaviour (DAS score > = 39).	Analyses of time trends revealed an early effect of Zuclopenthixol which could not be enhanced afterwards.	Dr. Glase- declared, Dr. Reis does not have conflicts of interest.<br>**Acknowledgements -** During the open and the withdrawal period this study was supported by the Bayer Vital GmbH, D 162,51368 Leverkusen, Germany.
Hanzel, T. E. *et al.* Results of Barbiturate Antiepileptic Drug Discontinuation on Antipsychotic Medication Dose in Individuals with Intellectual Disability, 2000, USA.	To explore the relationship between barbiturate AEDs (Phenobarbital), challenging behaviour and antipsychotic medication dose.<br><br>The authors hypothesized that antipsychotic medication was prescribed at doses higher than necessary to mitigate Phenobarbital behavioural side - effects. The authors further hypothesised that if Phenobarbital was discontinued, a decrease in challenging behaviour and antipsychotic medication would occur.	**Study Design –** Retrospective review of medical and behavioural records<br>**Population –** Adults with ID and epilepsy on an established regimen of Phenobarbital and an antipsychotic medication (Thioridazine or Chlorprothixene).<br>**Sample -** Convenience sample, (n = 5)<br>**Study Setting –** A public residential facility.	**1.** Over a 2 year period Phenobarbital was gradually reduced, discontinued and replaced with Carbamazepine or Valproic Acid. Concurrent tapering of antipsychotic medication also occurred. Medication weaning patterns, time taken for Phenobarbital reduction / discontinuation and dosage adjustments varied from patient to patient.<br><br>**2**. Behaviours that challenge were measured with a frequency count / partial interval recording and retrospectively analysed for time periods of 60 days:	Data supported the initial hypothesis.<br><br>Seizures for the 5 individuals were stable during the three measurement periods.<br><br>Phenobarbital was successfully discontinued for all 5 patients; 2 patients discontinued anti-psychotics entirely; the remaining 3 continued on antipsychotics at reduced doses.	Challenging behaviour collectively decreased by 81.5% after Phenobarbital discontinuation.<br><br>Mean antipsychotic medication dose decreased from 146 mg / day (SD = 98) to 106 mg / day (SD = 88) Chlorpromazine equivalence, with discontinuation in 2 cases.<br><br>Compared to the pre - Phenobarbital reduction period, challenging behaviour collectively decreased by 96.3% after the lowest antipsychotic dose was achieved; antipsychotic reduction was not achieved at the	**Funding –** Nil declared<br>**Conflict of Interest –** Nil declared<br>**Acknowledgements –** Nil declared<br>**Limitations -**<br>**1**. Inclusion and exclusion criteria for participants were unclear.<br><br>**2.** Small sample size (n = 5)<br>**3.** Staff were not blinded to medication changes, and inter - observer reliability checks were not made.<br><br>**4.** It is possible that Carbamazepine and Valproic Acid provided behavioural or psychiatric effects in addition to antiepileptic effects. Therefore the participants' challenging

eer Review Only

			• Before Phenobarbital reduction,<br>• After Phenobarbital discontinuation<br>• After the lowest antipsychotic medication dose had been achieved.<br>Behaviours that challenge were measured daily by staff trained by unit psychologists.		expense of behavioural deterioration.	behaviour may have represented an undiagnosed affective disorder which was successfully treated when Carbamazepine / Valproic Acid replaced Phenobarbital.
Holden, B. and Gitlesen, J. P. Psychotropic medication in adults with mental retardation: prevalence, and prescription practices. 2004, Norway.	The aim of the study was to investigate the prevalence psychotropic use in people with mental retardation living in a county in Norway, and to what extent best practice prescription guidelines were adhered to.	**Study Design** – cross-sectional study<br>**Population** – Adults with administratively defined mental retardation.<br>**Sample** – 300 Adults (>18 years) with mental retardation receiving at least a minimum of services from health or educational authorities.<br>**Study Setting** - Community setting in County of Hedmark, Norway.	A questionnaire designed for the study was distributed to each informant. Questions about (1) age, (2) gender, and (3) level of MR (as per the DSM IV) applied to all participants.<br><br>For participants using psychotropic medication, additional questions were asked: 4) Present medication(s). (5) When present medication(s) had been prescribed. (6) Psychiatric disorder(s)/ symptoms that had indicated each medication. (7) Problem behaviour(s). (8) Prescriber (e.g. psychiatrist, GP, consultation between GP and psychiatrist etc) (9) Whether effects of medication on symptoms/ behaviours	Forms for 294 participants (98%) were completed and returned anonymously. (Gender was missing for five participants.)<br><br>Age distribution of the total sample was: 18–30 years. There were 52.9% males (n=153) and 47.1% females (n=136).<br><br>110 (37.4%) used psychotropic medication. 25.9% (n=76) used 1 medication, 9.2% (n=27) used 2, and 2.4% (n=7) used 3 medications. Total number of prescriptions was 151, or 1.37 per member of the medication group.	19.4% of the participants (n=57) used traditional neuroleptics, 12.2% (n=36) used ‘‘second generation’’ neuroleptics, 8.5% (n=25) used SSRI antidepressants, 2.4% (n=7) used other antidepressants, 5.1% (n=15) used anticonvulsants, 1.7% (n=5) used anxiolytics, 1.4% (n=4) used mood stabilizers, and 0.7% (n=2) used stimulants.<br>- There was association between age and use of medication.<br>-On average, medications had been used for about five and a half years. Traditional neuroleptics had been used for 9 years, second generation neuroleptics had been used for just over two and a half years (P<0.001). There was	**Funding:** Nil declared.<br>**Conflict of Interest:** Nil declared.<br>**Acknowledgements:** Nil declared.<br>**Limitations:**<br>The local supervisors were familiar with the patients they were selecting for the study – potential for selection bias.<br>Details of participant/ caregiver consent were not given and it is unclear who designed the questionnaire for the study and whether the questionnaire was peer reviewed prior to use.<br>The study focused on community dwelling individuals with MR and their psychotropic use. The findings may not be generalizable to institutionalised adults/ prescribing practices may

			had been evaluated by formal data recording. (10) Whether side effects of medication had been evaluated by formal data recording. (11) Whether analyses of learning/ environmental factors possibly causing psychiatric symptoms/ behaviour problems had been conducted prior to medication. (12) Whether behavioural/environmental interventions had been attempted prior to medication.		little difference between duration of use of SSRIs and other anti-depressants. Eighty-two of 151 medications (54.3%), in 60 participants, were indicated by a psychiatric diagnosis/ and or problem behaviours. For 15.9% informants were unable to identify documented indications. 37.7% of the prescriptions were conducted by psychiatrists vs GPs. 73.7% (n=42) of psychiatrists' prescriptions were indicated by a diagnosis or symptom VS 42.6% (n=40) of GP prescriptions. 91.7% (22 out of 24) of medications prescribed without any indication had been prescribed by GPs. Prescribing differences: psychiatrists prescribed 57.9% of second generation neuroleptics versus 25.5% for GPs. GPs prescribed more antidepressants than psychiatrists, 29 versus 3.	differ in an inpatient setting. Several informants did not choose a psychiatric diagnosis on the questionnaire, but added a single symptom on their own. These symptoms, were added to the list of psychiatric indications by the authors, thus classifying them as psychiatric disorders rather than problem behaviours. This may have led to an incorrect estimation of participants' underlying diagnoses.
Janowsky, D. S., Barnhill, J., Shetty, M. &	To explore which doses of conventional ('typical') antipsychotic	**Study Design** - Retrospective review of records from 1990 -1997.	N/a	Analysis of reports of MDT Neuropsychiatric Behavioural Review	The mean initial treatment dose for high potency antipsychotic	**Funding –** Nil declared.

Davis, J. M., Minimally Effective Doses of Conventional Antipsychotic Medications Used to Treat Aggression, Self-Injurious and Destructive Behaviours in Mentally Retarded Adults, 2005, USA.	medications are necessary to maintain behavioural control in individuals with ID; and at which doses relapse occurs.	**Population** - Adults with ID who were receiving conventional ('typical') antipsychotic medications for the treatment of a variety of behavioural difficulties who were residents of a state facility who had been treated with 'typical' antipsychotic medications 1990 - 1997. **Sample** - 34 adults with ID who were receiving conventional ('typical') antipsychotic drugs for the treatment of a variety of behavioural difficulties. **Study Setting** - Murdoch Centre, a 580 – resident state facility for the treatment of individuals with ID; located in Butner, North Carolina, USA.		(NBR) conferences held at facility from 1990 – 1997 which included: **1.)** The diagnosis; **2.)** medications and changes in these medications; **3.)** Significant adverse reactions or side effects; **4.)** Significant laboratory tests and serum drug levels; **5.)** Weight changes; **6.)** Changes in target symptoms; **7.)** Changes in behavioural intervention plans; **8.)** Monitoring; **9.)** Progress toward goals; **10.)** Quantitative graphing of individual's target behaviours. Additionally, narratives were analysed for: **1.)** The initial stabilizing dose ; **2.)** The minimal fully effective dose; **3.)** The relapse -associated dose; **4.)** The reinstitution dose	medications was 11.4 ± 2.1 (mean ± SD) mg/d Haloperidol or Haloperidol equivalents in the case of Thiothixene and Loxapine. After the doses were lowered, the mean minimal fully effective dose of high potency antipsychotic medications necessary to control target symptoms was 5.9 ± 1.3 mg/d. All relapses were clinically significant.	**Conflict of Interest –** Nil declared. **Acknowledgments –** Nil declared. **Limitations** – The authors state that given the study design, intensification of symptoms may have represented a rebound phenomenon, rather than an unmasking of symptoms.<br><br>The authors stated that the timing of NBR intervals were irregular. Dosage reductions were not the same across individuals and the psychologists' evaluations were designed individually and were not standardized to any specific rating system.
Janowsky, D. S. *et al.,* Antipsychotic Withdrawal – Induced Relapse Predicts Future Relapses in	To investigate whether a previous unsuccessful antipsychotic drug withdrawal attempt predicts future drug withdrawal relapses in institutionalised	**Study Design** – 15 – year (1990-2005) retrospective review of quarterly Neuro-behavioural Review conference (NBR) reports. Review of such reports was carried out	Anti - psychotic drug withdrawal attempts.	Anti -psychotic drug withdrawal - induced relapse in those where an initial relapse had occurred previously.	49 of the 57 participants who had experienced an initial anti -psychotic drug withdrawal relapse had a subsequent withdrawal attempt. 14 (28.6%) experienced 1 relapse, 19 (38.7%)	**Funding** – Nil declared. **Acknowledgements** – Nil declared. **Conflict of Interest** – Nil declared. **Limitations** – Subject group consisted of a subgroup of

Institutionalized Adults With Severe Intellectual Disability, 2008, USA.	individuals with severe / profound ID.	more frequently than quarterly when available. **Population** – Persons with ID who had experienced an initial episode of deterioration of self-injurious behaviours, aggression, destruction / disruptive behaviour, or a combination of these behaviours during / after an attempt of first generation antipsychotic drug withdrawal. **Sample / Setting** - Residents of an intermediate - care facility for persons with ID (N = 57).			experienced 2, 10 (20.4%) experienced 3 and 4 (8.2%) experienced 4 additional relapses. 2 (4.1%) did not endure further relapse. The remaining 8 subjects remained on first generation anti-psychotics or were changed to second generation anti-psychotics and did not undergo further withdrawal attempts. By the end of the study period, only 4 (7%) of the 57 individuals were antipsychotic drug free. Results show that for individuals who experienced an initial intensification of maladaptive behaviours during withdrawal from first generation anti-psychotics, all but 2 participants had a subsequent relapse. Results also showed that once an antipsychotic medication was reinstated or had its dosage increased, the intensified symptoms declined over the course of 1 month. In summary, results suggest that following an initial relapse following a	institutionalised adults who had severe / profound ID. Assignment of a psychiatric diagnosis in this population is problematic. - Outcomes of individuals who were discharged from the care facility during the study period were not explored. Doses and the presence of concomitant medications were not held constant throughout the study period.

					antipsychotic drug withdrawal attempt, further relapses are likely following future attempts.	
Kastner, T., *et al.*, Long - Term Administration of Valproic Acid in the Treatment of Affective Symptoms in People with Mental Retardation, 1993, United States of America (USA).	To define diagnostic and treatment characteristics of affective symptoms in people with ID (response of symptoms to treatment with Valproic acid).	**Design** – Open trial. **Population** – 209 individuals with ID referred to a tertiary - care medical centre for evaluation of behavioural symptoms. **Sample** – 21 participants over a 2 - year period aged 8 - 40 years. **Setting** - Tertiary-care setting.	Interviews were conducted with parents for severity of illness. Clinicians rated patients' function using the Clinical Global Impression of Severity (CGI-S) Scale, observations in response to treatment and changes in psychotropic or anticonvulsant medication against the CGI-S. Valproate therapy initiated with endpoint behavioural remission. Drug levels in serum, complete blood counts, liver functioning and serum ammonium recorded.	18 patients completed the study, 78% responded favourably to Valproate therapy. 7 patients had a history of seizures, 4 were found to have suggested epilepsy following EEG. Patients with epilepsy improved more that patients without epilepsy. With the introduction of Valproic acid all original medication had been discontinued in 57% of patients.	The study suggested that affective symptoms, particularly in the presence of epilepsy are amenable to treatment with Valproic acid. Treatment with Valproic acid was associated with a reduction in major tranquilizers / other neuroleptic medications.	**Funding –** Nil declared. **Conflict of Interest-** Nil declared. **Acknowledgements** – Nil declared.
Kleijwegt, B. *et al.*, Support staff's perceptions of discontinuing antipsychotics in people with intellectual disabilities in residential care: A mixed - method study. 2019, Netherlands	To gain insight into support staff's perceptions of discontinuing antipsychotics in residential clients with intellectual disabilities.	**Study Design –** A mixed-methods design; four focus groups followed by a survey. **Population -** Support staff and 22 managers asked to recruit support staff. **Sample -** 11 managers recruited 29 support staff for focus groups and 187 of 347 surveys were completed.	Data gathered on: • Indications for the use of antipsychotics • Reasons for discontinuation. • Expectations regarding the discontinuation. • Attitude towards discontinuation. • Preconditions for discontinuation	Participants were willing to work towards lower dosages. In some cases, the initial indication for use antipsychotics was unknown and in the absence of evident behavioural or emotional disturbances, there were now no clear indications that antipsychotics should be used.	Antipsychotics were used mainly for managing behavioural or emotional disturbances and less for the treatment of psychiatric disorders. The Physician is seen as the leading professional in cases of discontinuation of antipsychotics. Discontinuation required a clear proactive plan.	**Funding** - Nil declared. **Conflict of Interest** - Nil declared. **Acknowledgments -** Nil declared.

For Peer Review

		**Study Setting –** Three organizations providing residential care for people with intellectual disabilities in the Netherlands.				
Niven, A. *et al.*, The use of psychotropic medication for people with intellectual disabilities and behaviours that challenge in the context of a community multidisciplinary team approach, 2017, Plymouth, UK.	To evaluate the use of psychotropic medications to manage behaviours that challenge in people with ID within the multidisciplinary context.	**Study Design -** Retrospective review of electronic healthcare records. **Population –** Adults with ID who exhibit behaviour that challenges and receive psychotropic medications. **Sample –** 106 clients who were open to the Community Intellectual Disabilities teams due to concerns surrounding behaviours that challenge. **Study Setting –** Community based Intellectual disability service in the UK.	Electronic records of each participant were examined to identify whether they had received any additional multidisciplinary team-based assessments.	In particular, the multidisciplinary assessments that were screened for included the following:<br><br>Nursing health assessments, alongside Psychiatric reviews, to ensure physical and mental health needs are identified and proactively addressed, including pain monitoring and other differential diagnoses.<br><br>Behavioural/psychological/functional analysis assessments.<br><br>Speech and language therapy assessments.<br><br>OT Assessments.	61 (58%) of participants were prescribed psychotropic medications with no diagnosed mental health condition. 56% (47) of clients were being prescribed antipsychotic medications, and 75% (34) of those taking antipsychotics were prescribed risperidone. The next highest prescribing rate was anxiolytic medications (29, 35%). 66% percent of participants had not had a nursing needs/physical health assessment completed which was documented on their electronic records within the last year. 72% had not had an initial behavioural assessment or functional analysis report referenced on electronic records. A greater number of clients had occupational therapy reports (occupation, 46%; sensory 41%)	**Funding** - Nil declared. **Conflict of Interest** - Nil declared. **Acknowledgments -** Nil declared.

For Peer Review Only

					and/or communication assessment (66%).	
O'Dwyer, M., Mestrovic, A. & Henman, M., Pharmacists' Medicines - Related Interventions for People with Intellectual Disabilities: A Narrative Review, 2015, Ireland.	**1.)** To explore what type of pharmaceutical care interventions were being undertaken for people with ID<br><br>**2.)** How pharmacists contributed to the care of people with ID as part of multidisciplinary teams.	**Study Design –** Narrative review. Systematic searches of telectronic databases were carried out, including: CINAHL, PubMed, Medline, Embase, Cochrane library, Science Direct and International Pharmaceutical Abstracts.<br>**Population –** N/A<br>**Sample -** N/A<br>**Study Setting –** N/A	N/A	**1.**) Pharmacist interventions.<br><br>**2.)** Pharmacists' collaboration in the provision of care.<br><br>**3.)** Qualitative studies relating to patient, carer and pharmacist views on the care of people with ID.	In one study, pharmacists provided a wide range of services but pharmaceutical care was not described. In total, only two intervention studies, 3 reports of pharmacists contributi ng to services for people with ID and one report of pharmacists' attitudes and opinions about caring for people with ID were published in this 20 year period. Most studies described pharmacists acting as part of an inter-disciplinary team, for patients within an institution or in sheltered accommodation. There were no studies of patients' / carers' views of pharmacists' contribution to care. The majority of studies identified originated in the USA or UK, suggesting that this research is of interest only in particular, high income countries, or that research in other countries is published in a non - English language	**Funding** - Nil declared.<br>**Conflict of Interest** - Nil declared.<br>**Acknowledgments -** MO'D would like to acknowledge funding received from a Trinity College Dublin Studentship.

					journals. The limited evidence available suggests that pharmacists' contributions offer a positive impact on the quality of medication use for people with ID, in collaboration with other health care professionals and carers.	
Patel N., Crimson L., Rush J., Frances A., Practitioner versus medication-expert opinion on psychiatric pharmacotherapy of mentally retarded patients with mental disorders. 2001, United States of America.	The prevalence of mental disorders among people with ID is increasing. This increased prevalence is associated with increased use of psychotropic medications in this patient cohort. There is a need for guidelines regarding the appropriate use of psychotropic medications in people with ID. The study sets out to investigate the degree to which practicing psychiatrists agree with such a set of guidelines.	The "Expert Consensus Guideline Series: Treatment of Psychiatric and Behavioural Problems in Mental Retardation" (PBPMR) provides a series of practical guidelines for practitioners in the treatment of mental illness in people with ID. During creation of these guidelines, a questionnaire was generated that was sent to experts on pharmacotherapy of mental illness in ID. This same survey was sent to psychiatric consultants at the Texas Department of Mental Health and Mental Retardation (TXMHMR) and to psychiatrists who spend a considerable amount of time treating patients with ID and co-existing mental disorders	N/a	N/a	37 (43.5%) of 85 practitioners completed the survey. Agreement - There was general agreement between medication experts and practitioners regarding preferred pharmacological treatments for specific diagnoses and target symptoms in patients. Valproate was considered treatment of choice for bipolar disorder while newer, atypical antipsychotics were regarded as the treatment of choice for self-injurious behaviour. Consensus was further evident in the preferred drug choice within this class which was risperidone. Divalproex and valproate were the preferred anticonvulsant-mood stabilisers for the treatment of self-	**Funding -** Nil declared. **Conflict of Interest -** Nil declared. **Acknowledgements -** Nil declared. **Limitations:** A Small number of responding practitioners (37 out of 85). Practitioner responses may not be a true reflection of practitioner practice.

For Peer Review Only

		(n=85). Survey analysis was based on using 95% confidence intervals (CI) and comparison of results for medication experts and practitioners was conducted.			injurious, aggressive or destructive behaviour in patients with coexisting epilepsy.<br>Differences - Practitioners favoured mirtazapine more than the medication experts in the treatment of self-injurious behaviour. Practitioners were more likely to prescribe psychotropic medications for the initial treatment of self-injurious or other disruptive behaviour.<br>Overall, general agreement was seen across the two groups.	
, Perry, B. I. *et al.,* Problem Behaviours and Psychotropic Medication Use in Intellectual Disability: a Multinational Cross - Sectional Survey, 2018, UK, China, Sri Lanka.	To determine the prevalence and possible associations of both psychotropic medication use and Problem Behaviours (PB's) from a multinational, cross - sectional sample of people with disorders of intellectual development (PWDID), recruited from community and hospital settings.	**Study Design -** A multinational, multi - setting, cross - sectional service evaluation survey and baseline audit. This included a semi - structured questionnaire completed by treating clinicians. The semi – structured questionnaire captured demographic details, prevalence rates of ID, psychotropic medication use and psychiatric co-morbidity.<br>**Population –** PWDID (adults) in the UK, China and Sri Lanka receiving psychotropic medications for potential PB's	**1.)** Samples from Sri Lanka and Hong Kong were grouped together, thus the sample was defined as 'UK' or 'non-UK'.<br><br>**2.)** A multinational, multi - setting, cross - sectional service evaluation survey and baseline audit was completed.<br><br>**3.)** Descriptive statistics were used to analyse demographic and prevalence data.<br><br>**4.)** Linear regression was used to analyse the association between	**1.** Prevalence of psychotropic medication use in PWDID<br><br>**2.** Association between psychotropic medication and co - morbid diagnoses<br><br>**3.** Association between co - morbid psychiatric diagnoses and type of PB<br><br>**4.** Association between psychotropic medication dosage and PB's	Psychotropic use was prevalent (90%), particularly antipsychotics (74%). Chi-squared analyses revealed no significant association between psychotropic use and gender / age (P > 0.05). Of the74% prescribed antipsychotics, 26% had documented evidence of psychotic features, 48% did not.<br><br>40% of the whole sample was prescribed mood stabilisers; 27% had a psychiatric diagnosis, 13% did not. Across all subgroups, there was no	**Funding** - Dr Benjamin Perry is supported by an NIHR academic Clinical Fellowship. Dr. Sherva Cooray receives funding from the Faculty of Intellectual Disability, Royal College of Psychiatrists, as chair of the ICD-11-Disorders of Intellectual Development Working Group.<br>**Conflict of Interest –** Nil declared<br>**Acknowledgements –** Nil declared<br>**Limitations –** Observational study; association (and not causation) between

For Peer R

		**Sample -** Total sample (n = 358); 65% being treated in inpatient facilities and 35% in outpatient clinics.<br>A sample of n = 247 (69% of the total sample) was collected from the UK, n = 61 from Hong Kong (17% of the total sample) and n = 49 from Sri Lanka (14% of the total sample).<br>**Study Setting -** Data was obtained from 15 hospital and community settings (UK / China / Sri Lanka), between 2013 and 2014.	psychotropic medication dosage and the presence and type of PB's.		statistically significant association between prescription of mood stabilisers and a psychiatric diagnosis.<br>The prevalence of PB's was 83%. There was no statistically significant association between psychotropic prescription and recorded psychiatric co -morbidity, suggesting prevalent 'off - label' use for PB's, or poor recording of psychiatric co -morbidity.<br>There was some evidence of possible diagnostic overshadowing (symptoms of psychiatric co -morbidity attributed to PB's). A higher dose of psychotropic medication was associated with aggression toward others (P = 0.03).	factors can only be demonstrated.<br>The sample analysed were of individuals in contact with DID services. It is possible that the reason for contact was secondary to PB's, which should be considered when analysing prevalence of use of psychotropic medication. Reverse causality may be an issue, e.g.: the finding that higher doses of psychotropics were prescribed for violence toward others may reflect paradoxical agitation caused by antipsychotics.
Radouco – Thomasa, M., Bolduca, M., Brissona, A., Brassarda,P., Fortiera, L., Thiviergea, J Pilot study on the use of psychotropic medication in persons with mental	To explore the status of psychotropic medication in persons with mental retardation (MR) 6 years after deinstitutionalization.	**Study Design -** Pilot study incorporating comparison of hospital charts of 2 patient groups.<br>**Population –** Persons with mental retardation that have been deinstitutionalised.<br>**Sample –**<br>1. Deinstitutionalization sample (D-sample): 59 patients (19 females, 40	1. D-sample: comparison of current psychotropic medication use of this sample with their psychotropic medication use 6 years previously.<br><br>2. In-sample: comparison of psychotropic medication use within this sample to the results of the D-sample comparison.	1. D-sample: 6 years post deinstitutionalisation; approximately the same percentage of patients remained on antipsychotic medications (AP= 72.8% pre VS. 71% post) and benzodiazepines (B=57.6% pre VS. 57.5% post). A three- fold increase occurred in antidepressant (AD)	The authors attributed the greater rate of reduction or withdrawal of all classes of psychotropic drugs in the In-sample VS. The D. sample to a concerted team effort at lowering medications. The authors admit that the groups were not controlled for confounding factors, e.g. age, sex, diagnoses etc;	**Funding:** Nil declared.<br>**Conflict of Interest -** Nil declared.<br>**Acknowledgements -** Nil declared.<br>**Limitations -**<br>The groups were not controlled for possible confounding factors (e.g. age/ sex, severity of MR, psychiatric diagnosis etc), which may affect findings.

retardation. 2004, Canada.		males) with a mean age of 58.7 (29–77). These patients were discharged from the hospital 6 years previously.<br>2. Current inpatient sample (In-sample): 25 patients (11 females, 14 males) with a mean age of 37.4 (19–66).<br>**Study Setting -** Centre Hospitalier Robert-Giffard- the mental hospital in Quebec City, Canada.		consumption (5% pre VS. 15% post). The use of mood regulators or anti-epileptics (M) increased from 37.2% to 49%.<br><br>2. In-sample: A general decrease of all types of psychotropic medication occurred (AP, B, AD, M) with complete withdrawal in a substantial number of patients (AP=36%, B=50%, AD=25%, M=40%).	the mean age difference between the two groups may also have been influential in this regard. Of note: the authors did not anticipate the finding of a greater rate of psychotropic medication reduction in the In-sample. These patients were admitted for acute issues which ordinarily would warrant de novo prescribing/ escalation of psychotropic drug doses. Additionally the authors commented on the greater number of persons with MR taking psychotropic drugs in their study (91.5%) VS. The mean in other American states (51.1%).	The number and identities of patients in each patient group (D-sample VS. In-sample) was not the same (bar one patient); this may have influenced the study results.
Ramerman, L., Hoekstra, P. J. & de Kuijper, G., Exploring Barriers & Facilitators in the Implementation & Use of Guideline Recommendations on Antipsychotic Drug Prescriptions for People with Intellectual	To explore barriers and facilitators in the implementation of guidelines concerning antipsychotic medication prescribing in people with ID.	Qualitative interviews were conducted with clinicians; four ID physicians, two psychiatrists and five behavioural scientists (n =11).<br>The interviews were semi-structured and guided by a questionnaire and participating clinicians were encouraged to explain their answers.	In depth interviews	Barriers and facilitators in the implementation of guidelines regarding antipsychotic medications for people with ID.	All 11 clinicians focused on prescribing antipsychotics for challenging behaviour, not for psychotic symptoms or schizophrenia. The rationale for such prescribing was due to the difficulty of such diagnoses in people with ID. A barrier identified was the setting culture in many of the organisations that care for people with ID. Mental disorders and	**Funding** – Nil declared.<br>**Conflict of Interest** – Nil declared.<br>**Acknowledgements** – Nil declared.<br>**Limitations** – Small sample size (n=11) and the limited involvement of clinicians' only and not all stake - holders.

For Peer Review

Disability, 2018, Netherlands.					behaviours that challenge are usually the responsibility of the ID physician / psychiatrist, despite recommendations encouraging involvement of the MDT. A barrier identified was the lack of ownership of guideline recommendations; Consensus occurred among all participants regarding a high administrative burden and the lack electronic patient records (potential for evaluation and monitoring of antipsychotics).	
Rasaratnam, R., Crouch, K. & Regan, A., Attitude to Medication of Parents / Primary Carers of People with Intellectual Disability, 2004, UK.	To investigate the influence of attitudes of care givers towards administering medications to people with ID.	**Study Design –** Interviews; the Rating of Attitude to Medication Scale (RAMS) interview schedule was used. The RAMS looked at separate areas of carers' attitudes surrounding medication, namely:<br>- Positive attitudes<br>- Negative attitudes<br>- Doctors' role in the medication regime<br>**Population -** Carers of service users with ID currently attending an outpatient clinic at a disability centre	-	-	A generic question from the questionnaire showed that there was a disproportionate number of parents displaying a negative attitude towards medications versus professional carers (46% vs. 11%). The results suggest that a carer's attitude towards giving medication may be a combination of subsets of beliefs. There was a significant difference in attitudes between parental carers and professional carers; professional carers having a more positive	**Funding –** Nil declared<br><br>**Conflict of Interest** – Nil declared.<br><br>**Acknowledgements** – Nil declared.

		**Sample** - 93 carers<br>**Study Setting –** An outpatient clinic at a disability centre<br>-			attitude which potentially yield more compliant medication regimes. The negative attitudes of parental carers may be a result of emotional factors or stigma associated with ID and psychiatric disorders. Many of the parents who are currently caring for adults with ID may not have received sufficient social support to enhance coping and positive attitudes. The results of this study suggests that more emphasis should be placed on working with family carers to improve compliance with medication.	
Ruedrich S, Swales P., Fossaceca C., Toliver J. & Rutkowski A., Effect of divalproex sodium on aggression and self-injurious behaviour in adults with intellectual disability: a retrospective review,. 1999, United States of America.	To evaluate the efficacy of valproate of aggressive or self-injurious behaviour in adults with ID.	**Study Design –** A retrospective review.<br>**Population –** Adults with intellectual disability who display severe, long-lasting behavioural problems.<br>**Sample** - 28 adults with a history of valproate use for reasons other than epilepsy.<br>**Study Setting –** 19 participants (68%) lived in a residential treatment centre and 9 (32%) lived in group home settings.	Treatment with divalproex sodium or Valproic acid for management of disruptive behaviours. The daily valproate dose ranged from 500-4000mg.	Monthly counts of aggression and self-injury were available for 17 of the 19 participants living in the residential centre. An objective measurement of target symptoms was available. Data was collected by staff members. For each patient, baseline behavioural counts were arranged for 2 months prior to commencement of valproate therapy and compared with counts averaging the final two months on valproate. All	Among the 17 participants in the residential centre where monthly counts of behaviour were available; there was a significant decrease in aggression and self-injury for 15 of these, while an increase was seen in 2. Taking the whole sample into account, 20 (71%) out of 28 were rated as demonstrating a moderate or great improvement while 6 (21%) showed a mild improvement. At the	**Funding** - Clinical research grant from Abbot Labs<br>**Conflict of Interest** - Nil declared.<br>**Acknowledgements:** Th e authors acknowledge the significant assistance by the staff of the Youngstoum Developmental Centre. Supported, in part, by clinical research grant from Abbot Laboratories.<br><br>**Limitations:** No control group.

For Peer Review

				28 participants were retrospectively rated with the Clinical Global Impression Severity (CGI-S) scale.	end of the study, 25 patients were still on valproate treatment.	Other medications were discontinued or reduced in some participants during the study.<br>Small sample size (n=28)
Ruedrich, S. L. *et al*., Atypical Antipsychotic Medication Improves Aggression, but not Self - Injurious Behaviour, in Adults with Intellectual Disabilities, 2008, USA.	To compare the effects of atypical antipsychotics with typical antipsychotics on self - injurious behaviour (SIB) and aggression.	**Study Design -** Retrospective review: Average monthly counts of aggression and self-injurious behaviour for 1 year of treatment with typical antipsychotics were compared with monthly averages for the next 1 year of treatment with atypical antipsychotics.<br>**Population –** Residents of a state centre with ID.<br>**Sample –** 31 adults with ID, treated for aggression and / or self -injurious behaviour with atypical antipsychotics.<br>**Study Setting –** State centre	1 year of treatment with atypical antipsychotics (Risperidone / Quetiapine / Olanzapine) along with the patients' existing medications.	Behavioural data, weight, glucose, cholesterol, other medication changes.	Subjects gained an average of 6.6lbs during the year of atypical treatment, but no significant changes in glucose or cholesterol were found. Subjects with aggression alone (N=14) had a statistically significant decrease in the number of aggressive acts per month during the year of atypical treatment (P=0.03); those with both aggression and SIB (N=12), or those with SIB alone (N=5) had no significant improvement. 27/31 subjects (87%) completed a full year of atypical antipsychotic treatment. 12/26 (46%) had typical antipsychotics discontinued within the year of atypical treatment; another 7/26 (27%) had their typical antipsychotic dose decreased. 23/31 trials involved risperidone; 7/31olanzapine; 1/31quetiapine. The findings suggest that atypical antipsychotics	**Funding** – Nil declared.<br>**Conflict of Interest** – Nil declared.<br>**Acknowledgements** – Nil declared.<br>**Limitations** – Names of typical psychotropics discontinued (bar Lithium) were not given. Neither atypical dose increases, nor typical antipsychotic withdrawal were done systematically. There was no randomization or placebo control. Although behavioural assessment counts of aggression and SIB were collected in a standardized manner, with oversight by psychology staff, no reliability measure or specific standardized instrument was utilized.

For Peer Review Only

					are well-tolerated and decrease the frequency of aggression in individuals with ID.	
Sabaawi, M., Singh, N. N. & De Leon, J., Guidelines for the Use of Clozapine in Individuals with Developmental Disabilities, 2006, USA.	To provide guidelines as a resource for psychiatrists and other physicians who treat mental illness and behaviours that challenge in individuals with developmental disabilities.	**Study Design -** narrative review article; three main sources: **Population -** individuals with developmental Disabilities. **Sample -** N/A **Study Setting –** USA.	The use of clozapine in individuals with developmental disabilities.	**1.)** Overview of necessary practical knowledge regarding side effects, dose and blood level considerations, and interactions with other medications, diet, tobacco and smoking. **2.)** Procedures for selecting individuals for clozapine therapy based on proper indications and contraindications for treatment. **3.)** Requirements regarding informed consent, dosage and special laboratory and clinical monitoring	Individuals with developmental disabilities who have failed behavioural and other drug treatments and exhibit behaviours that challenge that require pharmacological interventions appear to be good candidates for treatment with clozapine.	**Funding -** National Institute of Mental Health grant MH-47162 to George M. Simpson, M.D. and Richard C. Josiassen, Ph.D. Novartis Research Institute provided free medication for the clozapine double-blind study. **Conflict of Interest –** Jose de Leon - declared **Acknowledgments -** We thank Dr. Judy Singh and Ms. Rachel Myers for assistance in the preparation of the guidelines.
Sachdev, P., Drug - Induced Movement Disorders in Institutionalised Adults With Mental Retardation: Clinical Characteristics and Risk Factors, 1992, Australia.	To address potential risk factors and clinical presentation of tardive dyskinesia / extrapyramidal side effects in individuals with ID prescribed antipsychotics in current literature.	**Study Design –** **1.)** Historical information about participants was obtained from charts and medication records. **2.)** A detailed Inventory for Client and Agency Planning (ICAP) within the previous 12 months **3.)** A checklist of self–mutilation and stereotypic behaviours. **4.)** A neurological exam with the following: • Abnormal Involuntary	Neuroleptic drug use	**1.)** Tardive dyskinesia **2.)** Extrapyramidal side effects	32 (60.4%) of participants were currently on one or more neuroleptic medicines; all but 6 (11.3%) had been on neuroleptic medications at some point of their stay at the institution. Using a global AIMS score of 2 or more as a diagnosis of TD, 16 (34%) of the 47 participants who had been exposed to neuroleptics had TD. 5 of these participants had had their neuroleptic	**Funding –** Nil declared. **Conflict of Interest –** Nil declared **Acknowledgements –** Nil declared

		Movements Scale (AIMS)<br>• Scale for Extrapyramidal Side Effects and Akathisia Rating Scale.<br>**5.)** A 10 - minute video of drug-induced movement disorders.<br>**Population:** Institutionalised adults with ID<br>**Sample –** N=53<br>**Study Setting –** Institution			medication stopped in the preceding 3 months; 3 of these had no TD at the time of cessation which suggests withdrawal induced TD. The other 2 had had their medication reduced in the preceding 3 months due to the emergence of dyskinetic movements.	
Schwarz, V. *et al.*, Therapeutic Drug Monitoring of Zuclopenthixol in a Double - Blind Placebo-Controlled Discontinuation Study in Adults with Intellectual Disabilities & Aggressive Behaviour, 2014, Germany.	To assess the relationship between oral dose of Zuclopenthixol, serum concentration, and therapeutic efficacy in patients with intellectual disability showing aggressive disruptive behaviour.	**Design** - Multicentre, double blind, randomized trial with placebo - controlled parallel group comparison with a withdrawal design.<br>**Population** – Patients with ID between 18 and 50 years with aggressive behaviour.<br>**Sample** – 49 people with ID.<br>**Study Setting** - 10 centres in Germany.	Drug dosage, Zuclopenthixol serum levels and clinical assessment on efficacy using the Disability Assessment Schedule (DAS) and the Modified Overt Aggression Scale (MOAS) were analysed.	10 patients did either not respond to the medication or dropped out before randomization.<br>The associations between dosage, serum concentration and clinical results indicated no comprehensive patterns.	Subjects receiving placebo after randomization showed increased aggressive behaviour.<br>A dose reduction was associated with a clinical improvement.	**Funding** – Nil declared.<br>**Conflict of Interest** – Haessler, F. - declared Hiemke, C. - declared Glaser, T, - declared. Other authors: no conflicts of interest reported.<br>**Acknowledgements** - This study was supported by the Bayer Vital GmbH, D 162, 51368 Leverkusen, Germany.
Shankar, R. *et al.*, A Structured Programme to Withdraw Antipsychotics	To use a structured pathway to achieve withdrawal of antipsychotics among adults with ID. This pathway involved people	**Study Design** - The authors commenced the withdrawal programme in several steps:<br>Step 1:	Drug withdrawal as per developed structured pathway.	Dose reduction and / or withdrawal	Of the 71 adults with ID who were assessed for withdrawal; 33 (46.5%) achieved complete cessation of antipsychotics, 8 (11.3%)	**Conflict of Interest / Funding** RS - declared. MW,SD,RG,EC,CP, GP, AP, EW, RL, SA, RS and RA do not report any conflict of interest.

Among Adults with Intellectual Disabilities: The Cornwall Experience, 2019, UK.	with ID, their carers, general practitioners (GP's), community learning disability team members (CLDT) and pharmacists.	Presentation to local GP's describing the authors' proposal to withdraw antipsychotic medication.<br>Step 2: Involving all stakeholders<br>Step 3: Secondary care Authors regularly involved CLDT members to discuss the withdrawal strategy.<br>Step 4: Development of tools to facilitate the withdrawal process.<br>Step 5: Assessment of patients for withdrawal<br>Step 6: Follow-up & contingency plan<br>**Population –** Community based adults with ID, their carers, GP's, CLDT and pharmacists.<br>**Sample –** 71 adults with ID.<br>**Study Setting -** Community			achieved over a 50% dose reduction, 18 (25%) achieved less than a 50% reduction and withdrawal failed in 7 (9.8%). The remaining 5 (7%) were identified as needing a more appropriate placement before withdrawal could be considered. The success rate for complete withdrawal of antipsychotics is similar to the rates of previous similar studies. The failure rate was low at 9.8%. The authors concluded that withdrawal / reduction is achievable through involvement of all stakeholders.	
Sheehan, R. & Hassiotis, A., Reduction or Discontinuatio n of Antipsychotics for Challenging Behaviour in Adults with Intellectual Disability: a Systematic Review, 2017, England (UK).	To systematically review the available literature.	**Study Design -** A systematic review.<br>**Population -** All study designs of participants with intellectual disability over 18 conducted in community or institutional setting who must have been taking an antipsychotic regularly for at least 12 weeks before the intervention (reduction or discontinuation of	Studies that addressed an intervention for the reduction or discontinuation of antipsychotic medication.	**1.)** Discontinuation of anti - psychotics.<br><br>**2.)** Maintenance of a reduced dose of anti-psychotics.<br><br>(The proportion of participants maintained on a reduced dose was between 19% and 83%. Discontinuation of antipsychotics ranged from 4% to 74%. The	Discontinuation practices included dose - reduction programmes that were under clinician control. There was a transient increase in dyskinesia that accompanied antipsychotic reduction or discontinuation that persisted for several months and returned to baseline over longer follow - up periods.	**Funding –** Nil declared.<br>**Conflict of Interest -** RS declares no competing interests. AH was a member of the NICE Guideline 'Challenging behaviour and learning disabilities: prevention and interventions for people with learning disabilities whose behaviour challenges' (NG11).

For Peer Review O

		antipsychotic medication). **Sample -** 21 studies. **Study Setting –** International published literature in 5 databases (PsycInfo, Medline, Embase, Cinahl, Cochrane).		proportion unsuccessful in attempts to reduce or discontinue antipsychotics was between 0% and 96 %.)		**Acknowledgements –** Nil declared.
Sheehan, R. *et al.,* A Structured Medication Review Tool to Promote Psychotropic Medication Optimisation for Adults with Intellectual Disability: Feasibility Study, 2019, England (UK).	To investigate the feasibility of delivering structured psychotropic medication review in community services for adults with ID.	**Design** – Single - arm feasibility study conducted over a 6 - month period. **Population** – 15 psychiatrists working with adults with an ID and 94 adults with an ID who had been prescribed psychotropic medication. **Sample** – 15 psychiatrists, 79 adults with an ID. **Setting** - Specialist community ID teams in England.	A structured web -based psychotropic medication review tool was made available for use by psychiatrists in routine clinic appointments, (the HealthTracker -based structured medication review (HT - SMR): comprising measures of therapeutic benefit and adverse side –effects). A summary measure of medication effectiveness was graphically presented to aid discussion and decision-making.	The HealthTracker - imputed MEI was tested as a potential future outcome measure. The HT-SMR was broadly acceptable; suggestions were made for adaptations to improve integration with existing information technology systems and to enhance patient involvement. The structured medication review tool demonstrates that it would be feasible to test outcomes in a definitive trial.	There is a need to improve the quality of psychotropic medication use in people with ID, despite consensus guidelines of good practice.	**Funding** – Sheehan, R. is funded by a National Institute for Health Research (NIHR) Doctoral Research Fellowship. **Conflict of Interest** – Fiori, F. - declared. Santosh, P. declared. **Acknowledgements** - The clinical services staff who recruited participants and completed the HT-SMR, members of the Camden Synergy service user consultation group (Jill Huntesmith and Jackie McMorrow), and the research participants and their carers.
Sheehan, R., Hassiotis, A., Strydom, A. & Morant, N., Experiences of Psychotropic Medication Use and Decision - Making for Adults with	To explore experiences of psychotropic medication use among people with ID and their carers. The study also focused on how medication – related decisions are made.	**Study Design -** Qualitative; semi - structured Interviews with subsequent thematic analysis of data collected. **Population -** People with ID and their carers. **Sample -** 14 adults with ID, 12 family carers and 12 paid carers.	N /a	The relational and power dynamics underpinning decision making regarding psychotropic medication.	**1.)** People with ID reported being highly compliant with psychotropic medication. Compliance was based largely on an unquestioned view of medication as important / necessary and belief in the authority of the psychiatrist. Although they sometimes	**Funding** - This study was funded by a Doctoral Research Fellowship awarded to Sheehan, R. from the National Institute for Health Research (NIHR). **Conflict of Interest -** Authors declared no competing interests.

Intellectual Disability: a Multi-stakeholder Qualitative Study in the UK, 2019, UK.		**Study Setting –** Specialist psychiatry services, community groups, care providers and training organisations in the UK.			experienced medication negatively, they were generally not aware of their right to be involved in medication – related decisions. Additionally, people with ID reported having few opportunities to become involved in the medication decision - making process. **2.)** Paid and family carers reported undertaking a number of medication - related activities. Their 'front-line' status and longevity of relationships meant that carers felt they possessed important forms of knowledge relevant to medication - related decisions. Both groups of carers valued decision making in which they felt they had a voice and a genuine role. **3.)** While some participants in each group described making joint decisions about medication with psychiatrists, lack of involvement was often described. This took three forms in participants' accounts: 1.) being uninformed of	**Acknowledgements** - The authors thanked those who agreed to take part in the study, the organisations and individuals that assisted with recruitment, and Restu Handoyo, who contributed to the analysis. They also thanked members of the service user consultation group, Jackie McMorrow and Jill Huntesmith. **Limitations -** Those with limited or no verbal ability were unable to take part in the study.

					important facts, 2.) insufficiently included in discussions and 3.) Lacking influence to shape decisions. Participants described efforts to democratise the decision - making process by gathering information, acting to disrupt perceived power asymmetries and attempting to prove their credibility as a valid decision- making partners.	
Smith, C. *et al.,* Sedation Effects on Responsivenes s: Evaluating the Reduction of Antipsychotic Medication in People with Intellectual Disability Using a Conditional Probability Approach, 2002, Wales & England (UK)	To explore staff / resident interaction and behavioural engagement as a measure of responsiveness during dug withdrawal.	**Design** - Participants were allocated randomly to experimental (n = 36) and control groups (n = 20). Each participant was studied for 6 months. **Population** - 67 potential participants currently taking antipsychotic medication for a behavioural problem. **Sample** – 56 participated. **Setting** – institutional or community residential homes.	Baseline assessments (month 1) were followed by 4 monthly drug reduction stages of 25% of the baseline Chlorpromazine equivalent dose (months 2 - 5). Month 6 was included to allow for any delayed changes in behaviour after drug cessation.	Drug reduction group who underwent at least 75% reduction, had a lower baseline mean than the control and failure groups; those who underwent 100% drug reduction, had a similar baseline mean to the control and failure groups.	Most frequently prescribed: Thioridazine (12%), Haloperidol (23%) and Chlorpromazine (14%). 20% were prescribed 2 or more antipsychotic drugs, excluding PRN medications; 79% had been prescribed the same drugs for 5 years or more. The mean daily Chlorpromazine equivalent dose per participant was 372mg (range = 20-4067mg, SD = 613mg).	**Funding** – support from the Wellcome Trust. **Conflict of Interest** – Nil declared. **Acknowledgements** - This analysis was undertaken as part of a programme of work funded by the Wellcome Trust.
Sohanpal, S. K. *et al.*, The effectiveness of antidepressant medication in	To systematically review the literature regarding the effectiveness of antidepressant medications for the management of	**Study Design** – Systematic review. **Population** – Adults with ID exhibiting a behaviour problem. **Sample –** N/A	N/A	Any outcome related to the behaviour before and after the intervention had to be stated. The various studies included	The existing evidence on the use of antidepressants for the management of behaviour problems in adults with ID is scant.	**Funding** – This study was funded by the Big Lottery Fund and was administered by MENCAP.

the management of behaviour problems in adults with intellectual disabilities: a systematic review, 2007, Birmingham, UK.	behaviour problems in adults with ID.	**Study Setting** – N/A		the following outcome measures:<br>(a) Aberrant behaviour checklist (ABC)<br>(b) The Handicaps, Behaviour and Skills schedule (HBSs)<br>(c) Standard behavioural observation techniques.<br>(d)Standardized Modified Overt Aggression Scale.<br>Amongst other behavioural outcome measures.	Responses to SSRIs across the literature varied; some studies reported favourable responses while others reported negatives effects. Reported improvements were largely reported in SIB and preservative/compulsive behaviours.<br>Based on the poor-quality evidence, the authors concluded that antidepressants, particularly SSRIs, improve aggression, SIB and other behaviour problems on average in less than 50% of cases and the rest show either no improvement or deterioration.	**Conflict of Interest** – Nil declared.<br>**Acknowledgements –** as above.
Song, M., Ware, R., Doan, T. N., Harley, D., Psychotropic medication use in adults with intellectual disability in Queensland, Australia, from 1999 to 2015: a cohort study.	To investigate psychotropic medication use, commencement and cessation, and associated factors in adults with ID.	**Study Design** – A Cohort study design.<br>**Population** – Adults with ID.<br>**Sample –** Adults with ID living in 24 hour supported accommodation with Endeavour Foundation; Queensland's major service provider to adults with ID. Adults were originally enrolled as part of a cluster RCT (1999 - 2001) of the comprehensive health	Review of CHAP booklets and baseline carer questionnaires. Data was coded once extracted. Analysis was performed using Stata statistical software.	Of the 453 RCT participants recruited in 1999, 138 (30%) agreed to participate in this follow-up cohort study in 2016. There were 697 reviews completed in total, with 138, 93, 111 and 92 participants reviewed in the periods 1999–2001, 2002–2006, 2007–2011 and 2012–2015, respectively. The median number of reviews completed per person was 5.	At baseline, in 2002–2006, 2007–2011 and 2012–2015, respectively, 43%, 51%, 53% and 54% of the participants were prescribed psychotropic medications.<br>Antipsychotics (22–28%) and antidepressants (17–36%) were the most frequently used psychotropics.<br>Psychotropic medications were commenced in 18, 14, and 9% of the participants between	**Funding -** Nil declared.<br>**Conflict of interest -** Nil declared.<br>**Acknowledgements - Nil** declared.<br>**Limitations** - Limitations were acknowledged by the authors. The frequency of reviews varied among individuals, leading to difference in the number of participants reviewed in the four time periods. A validated instrument for

2020, Australia.		assessment programme (CHAP). The study cohort was recruited from the RCT group. 138 of the RCT participants agreed to participate in this study.<br>**Study Setting** - 24 hour supported accommodation with Endeavour Foundation.			consecutive periods of 1999–2001 to 2002–2006, 2002–2006 to 2007–2011 and 2007–2011 to 2012–2015. Medications were ceased in 15, 7 and 7%, respectively. Challenging behaviour and psychiatric illness were positively associated with use of psychotropics. Challenging behaviour and psychiatric illness were positively associated with change in psychotropic use. Presence of challenging behaviour (consistent or new) was positively associated with ongoing use or commencement of psychotropics. Psychotropics were prescribed without diagnosis of psychiatric illness in 35% of participants, and antipsychotics were prescribed without diagnosis of psychosis in 16%	measuring behaviours that challenge was not used; challenging behaviour was determined based on a checklist and support staff reports. It was at the support staff discretion to determine which behaviours qualified as challenging. Psychiatric illness was assumed to be chronic. The study did not consider changes in drug, dosage and periods off medication.
Tan, X., , S. R. Marshall, V. D., Balkrishnan, R., Patel, I., Dunn, B. J., Chang, J. and Erickson. Psychotropic	The study investigated (a) oral psychotropic medication adherence among community-based adults with developmental disabilities (DD) and	**Study Design** - Retrospective cohort study which analysed/ reviewed Marketscan® Multi-State Medicaid Database data, from	N/A	3 main outcome measures were measured:<br><br>1. Medication adherence.<br>2. Healthcare utilization	Among all patients, antidepressants and antipsychotics were the most commonly used drugs (73.7% and 54.0%, respectively). Proportion of Days Covered (PDC)	**Funding** - Nil declared.<br>**Conflict of Interest** - Nil declared.<br>**Acknowledgments** - Nil declared.

For Peer Review Only

Medication Adherence among Community-Based Individuals with Developmental Disabilities and Mental Illness. 2015, USA.	mental illness, and (b) assessed the association between psychotropic medication adherence and healthcare utilization/ costs.	January 2003 – December 2007.<br>**Population –** Individuals with a dual diagnosis of developmental disabilities and mental illness.<br>**Sample -** 3,905 adults aged between 18-64 with a dual diagnosis of developmental disability and mental illness and newly initiated psychotropic medication.<br>**Study Setting –** Community based.		3. Health costs	was measured to estimate psychotropic medication adherence. The mean PDC was 0.86, and the non-adherence rate was 25.7%.<br>African Americans were 41% less likely to adhere to psychotropic medications than Caucasians ($p<0.01$), while males were more likely to adhere than females [OR] =1.23, $p<0.01$). Having GERD was significantly associated with worse adherence (OR=0.78, $p<0.01$). The odds of adherence among capitated plan enrolees were 29% lower than those of FFS enrolees ($p<0.01$).<br>Factors associated with psychotropic medication adherence: those who used antidepressants or antipsychotics had higher adherence rates than those who did not (OR=1.66, $p<0.01$ and OR=1.85, $p<0.01$, respectively), after controlling for other factors. Better psychotropic medication adherence was related to reductions in ER visits,	**Limitations -** Limitations were acknowledged by the authors.<br>A retrospective cohort study prevents causal inference, so association can only be claimed.<br>Caregivers' perceptions and willingness to administer medications was not explored.<br>The claims and expenditure data in Medicaid capitated plans may not be as accurately documented as those in FFS plans<br>Lack of accurate indications for the psychotropic drugs used, the authors could not guarantee that psychotropics were prescribed for mental illness/ behavioural issues/ other conditions.<br>This study relied on the following assumptions in using administrative claims data: (a) The pharmacies billed their services in an accurate/ timely manner; (b) all of the psychotropic drugs were covered by Medicaid; (c) the drugs filled were the drugs actually taken by the patients.

					suggesting better utilization outcomes. Capitated plan enrolees, compared with Fee for Services (FFS) enrolees, had 89% fewer psychiatric outpatient visits, 54% fewer outpatient visits, 23% more ER visits, and 70% lower total costs associated with medical services (all p<0.01).	The study population were Medicaid enrolees who were new users of psychotropic medications.
Troisi, A., Vicario, E., Nuccetelli, N. Ciani, A. Posini, A., Effects of Fluoxetine on Aggressive Behavior of Adult Inpatients with Mental Retardation and Epilepsy, 1995, Italy.	To investigate the effects of fluoxetine on aggressive behaviour in adults with ID.	**Study Design -** An alternating control-treatment-control design was adopted. **Population –** Adults with intellectual disability with a history of epilepsy and current or recent aggressive behaviour. **Sample -** 19 in-patients who had been institutionalised for a long period of time. **Study Setting –** Participants were recruited from intellectual disability clinics.	Treatment with 20mg fluoxetine daily.	Changes in aggressive behaviour - which was measured using the Modified Overt Aggression Scale (MOAS).	-Fluoxetine treatment was associated with changes in the MOAS ratings of total aggression, verbal aggression and self-aggression; the ratings for all categories of aggression increased during drug treatment and declined following drug withdrawal. -9 patients (group 1) displayed an increase in aggression, 8 (group 2) displayed no significant change and 2 (group 3) showed a reduction in aggressive behaviour. -After drug withdrawal, the MOAS ratings of group 1 and 2 decreased sharply and fell below pre-treatment levels. In contrast, aggressive behaviour in group 3	**Funding -** Nil declared. **Conflict of Interest -** Nil declared. **Acknowledgements:** We thank Gary Tollefson, MD, for his useful comments on an earlier version of this paper. **Limitations:** -Variation of treatment phases (4-14 weeks). -Not double-blinded. -No placebo. -Small sample size (n=19) -MOAS places strong emphasis on overt aggressive behaviour and leaves little room for subjective interpretation. -Patients are taking an array of other medications including anti-epileptics and neuroleptics.

Peer Review Only

					increased and surpassed pre-treatment levels.<br><br>The apparent association between fluoxetine and levels of increased aggression may be explained by an adverse effects due to drug interactions or fluoxetine overmedication and a serotonin mediated effect on regulation of aggression.	
Tsakanikos E., Costello, H., Holt, G., Sturmey, P. & Bouras, N., Behaviour Management Problems as Predictors of Psychotropic Medication and Use of Psychiatric Services in Adults with Autism, 2006, UK.	To evaluate behaviour problems as predictors of psychotropic medication use, psychiatric service use and inpatient admission in adults with Pervasive Developmental Disorder (PDD) and ID living in community settings.	**Design** - case control cohort study<br>**Population** – **Inclusion criteria:** Individuals with ID (F70 — 73), presence of behaviour problems and a PDD diagnosis (for the PDD group).<br>**Exclusion criteria:** Presence of an additional psychiatric disorder, such as depression, schizophrenia spectrum, anxiety, personality disorder etc.<br>**Sample** -168; 69 adults with PDD and 99 controls (i.e. adults with ID but without PDD) matched in age.<br>**Study Setting** - East London, community setting.	N/a	**1.)** Primary outcomes (for psychotropic medications) - Medication was coded into the following categories: 'anti-psychotics', 'sedatives', 'anti-convulsants', 'PRN only', 'combinations' and 'none'.<br>**2.)** Secondary outcomes - Disability Assessment Schedule (DAS); psychiatric consultation and hospital admission.	**1.)** Overall, a significantly larger proportion of people with PDD received psychotropic medication (v2 = 3.78, p < .05) as compared to controls.<br>**2.)** People with PDD were more likely to receive anti-psychotics (v2 = 7.77, p < .005) than controls.<br>**3.)** In addition, a significantly smaller proportion of people with PDD took anti-convulsants (v2 = 8.11, p < .01).<br>**4.)** There were no statistically significant differences between groups with respect to combinations of medications, sedatives and PRN medication.	**Funding –** Nil declared.<br>**Conflict of Interest –** Nil declared.<br>**Acknowledgments –** thanks were given to the participant service users and their carers and 3 anonymous reviewers<br>**Limitations** – The range of variables included in the regression analyses was limited to client maladaptive behaviours measured on the DAS. Likewise, variables related to services and attitudes among the professionals were not included. The study focused on a limited range of psychiatric services.

For Peer Review

Tyrer *et al.*, Risperidone, Haloperidol and Placebo in the Treatment of Aggressive Challenging Behaviour in Patients with Intellectual Disability: A Randomised Controlled Trial. 2008, England & Wales (UK) & Australia.	To compare flexible doses of haloperidol, risperidone and placebo in the treatment of aggressive challenging behaviour.	**Study Design –** a three-arm, parallel-group pragmatic trial of placebo, haloperidol, and risperidone with balanced randomisation, but no stratification, into each arm, and blind assessments of outcome at 4, 12, and 26 weeks after randomisation. Clinical assessments of aggression, aberrant behaviour, quality of life, adverse drug effects, and carer uplift and burden, together with total costs, were recorded at 4, 12, and 26 weeks. Change in aggression recorded with the modified overt aggression scale (MOAS). **Study Population** – all patients being treated by services for intellectual disability (N=180). **Study Sample** – 86 non - psychotic patients presenting with aggressive challenging behaviour, randomly assigned to haloperidol (n=28), risperidone (n=29), or placebo (n=29). **Study Setting** – ten centres in England and Wales, and one in Queensland, Australia. All patients except one (in	Participants with recent challenging behaviour and aggression with a total MOAS score of at least 4 in the last 7 days were assigned to placebo, risperidone, or haloperidol groups.	A reduction in aggression was noted with all treatments after 4 weeks, however, the greatest decrease was with placebo.	No differences between groups were noted in terms of aberrant behaviour, quality of life, general improvement, effect on carers, and adverse drug effects.	**Funding -** National Coordinating Centre for Health Technology Assessment (NCCHTA). **Conflict of interest** – Nil declared. **Acknowledgements** – the NCCHTA for their funding. The Mental Health Research Network, the University of Queensland for hosting the trial. Freya Tyrer for background data; the UK charity MenCap for supporting and promoting the trial; all individuals involved in this study. Laure Lenôtre, Joanna Watson, and Jill Thompson who assisted in recruitment; the consultants who took part in recruitment, especially Angela Hassiotis and S Ganeshanathan; Ben Thrift and Malcolm Walker; the Trial Steering Committee (Sheila Hollins, Stephen Tyrer, and Angela Hassiotis), the Data Monitoring and Ethics Committee (William Fraser, Tony Johnson & Deborah Rutter) and people with intellectual disability and their carers who participated in this trial.

For Peer Review Only

		hospital) were recruited from community settings.				The medication was provided by Janssen -Cilag, £6000 spent on preparation of the medication by the pharmacy staff of the trial centre pharmacy.
Valdovinos, M. G., Henninger-McMahon, M., Schieber, E., Beard, L., Conley, B., Haas, A., Assessing the impact of psychotropic medication changes on challenging behaviour of individuals with intellectual disabilities., 2016, USA	To examine how the use of/changes in, multiple psychotropic medications (combination treatment) impacts behaviours that challenge in individuals with ID.	**Study Design –** The current study followed eight individuals over a two-year period as they experienced changes in their psychotropic medication regimens. During that time, data from functional analyses and indirect assessments of challenging behaviour were collected.<br>**Population –** Adults with ID and challenging behaviour.<br>**Sample -** 8 adults with ID and challenging behaviour. Individuals were identified by agencies that provided care to persons with ID.<br>**Study Setting** – N/A	1. Initially direct care staff were administered two questionnaires during the initial screening. The Questions about behavioural function (QABF) form and the ABC-C questionnaire.<br>2. Administration of a Functional Assessment Interview (FAI) was conducted with staff to assess the individual's history of challenging behaviour, conditions under which the challenging behaviour was thought to be more likely to occur, and to identify any potential stimuli that were hypothesized to function as reinforcers/causes of challenging behaviour.<br>3. Authors were notified when a participant experienced a medication change.<br>4. Video recordings of functional analysis (FA)'s were scored for the occurrence of challenging behaviour as	Data from functional assessments was used to assess hypotheses about challenging behaviour in participants with ID after psychotropic medication changes (see methodology).	FA outcomes were sensitive to psychotropic medication changes. This suggests a need for continued behavioural assessment to inform medication practices. These changes were either changes in the rate of responding and/or the type or level of behaviour which occurred.<br><br>Specific findings: Of the 8 participants in the study, 5 participants' behaviours varied in rate of responding in FAs across all medication changes. 3 participants' FAs produced changes in outcomes; however, those changes were not consistent across all medication changes, that is, not every medication change yielded different outcomes from previous assessments.<br><br>The QABF outcomes were inconsistent with the FA outcomes. This	**Funding-** National Institute of Child Health and Human Development [grant number 1R15HD072497-01].<br>**Conflict of Interest -** Nil declared.<br>**Acknowledgements -** Nil declared.<br>**Limitations:**<br>Limitations were acknowledged by the authors<br>The small sample size limits and generalizability. There were many false positive functions identified by the QABF, it is difficult to consider it a valid measure of medication effects on behavioural function. The irritability scores on the ABC-C fluctuated and the severity reflected in those scores did not always match the frequency of behaviour observed within the FAs. staff who did not receive

			operationally defined by trained, undergraduate research assistants using Noldus Information Technology's software, The Observer® XT.		was surprising because research has demonstrated that QABF results are generally consistent with FA outcomes	A significant limitation of this study was the lack of inter-observer agreement (IOA) on staff collected data (i.e. QABF and ABC-C); (staff training might be an issue)
Young, N., J. & Findling, R. L., Pharmacotherapy for Mental Health Problems in People with Intellectual Disability, 2016, USA.	To review current evidence-based pharmacotherapy options / recent updates to guide clinicians in their medication management plans for individuals with an ID and concurrent mental health problems.	**Study Design –** Review, primarily focused on randomized placebo - controlled trials (RPCTs) with a sample size of 10 or more within the entire study. Genetic and other syndromes commonly associated with PWID (People with Intellectual Disabilities) were also included. **Population** – N/A **Sample** – N/A **Study Setting** – N/A	N/a	The search focused on studies that targeted mental health problems with pharmacotherapy and excluded studies solely focused on other medical problems.	Antipsychotics, particularly Risperidone, effective in reducing problem behaviours in children with ID; evidence in adults is inconclusive. Methylphenidate and alpha-agonists appear effective in reducing ADHD symptoms. Lithium may be effective in reducing aggression. Limited evidence for antiepileptic drugs, anxiolytics, and naltrexone for problem behaviours. Antidepressants may be poorly tolerated and may not be effective in reducing repetitive / stereotypic behaviours. Glutamatergic and GABAergic agents for fragile X syndrome and acetylcholinesterase inhibitors for Down's syndrome failed to show efficacy. Growth hormone treatment might improve cognition	**Funding –** Nil declared. **Conflicts of Interest** - Young N., J. had no conflicts of interest. Findling, R. L., declared. **Acknowledgements –** The authors would like to thank Dr James Harris, and Katherine Kendrick.

For Peer Review Only

					and behaviour in Prader – Willi syndrome. Oxytocin trials for behaviours are inconclusive. Melatonin appears to improve sleep. Most trials of dietary supplements did not show benefits.	
Zaala, R. J., Ebbersb, S., Bormsc, M., De Koningd, B., Mombarge, E., Oomsf, P., Vollaardg, H.,Van Den Bemta, P. & Evenhuis, H. M., Medication Review Using a Systematic Tool to Reduce Inappropriate Prescribing (STRIP) in Adults with an Intellectual Disability: A Pilot Study, 2016, Netherlands	**1.)** To evaluate the process of medication review using STRIP in adults with an ID living in a residential care setting.<br><br>**2.)** The identification of drug - related problems.	**Study Design –** Feasibility study.<br>**Population –** Adults with an ID in residential care.<br>**Sample -** 27 reviews<br>**Study Setting –** 3 Dutch residential care organisations for adults with an ID.<br>In the Netherlands, the legal representative of a client with ID is required to give consent to any changes in medical treatment. Ideally the client would also agree.	The Systematic Tool to Reduce Inappropriate Prescribing (STRIP) was used in a medication review performed by the investigator with a pharmacist.	**1.)** Time -investments of the investigator and the pharmacist were described.<br>**2.)** The proportion of reviews in which a client and / or their legal representative participat ed was calculated.<br>**3.)** The proportion of professional caregivers that participated.<br>**4.)** The proportion of clients with at least one drug-related problem was calculated.<br>**5.)** Additional outcomes in this evaluation were:<br>• The number and types of drug - related problems identified.<br>• The implementation rate of suggested changes in pharmacotherapy<br>• The net savings on direct pharmaceutical costs<br>• The net savings of interventions that	**1.)**<br>27 reviews were perform ed.<br>For each client included in the review, at least one drug-related problem was identified. In total 127 drug - related problems were identified.<br>**2.)** During the review process, 4 potentially appropriate indicated drugs were identified by START in 4 clients (15% of the study population). 18 potentially inappropriate or unnecessary drugs were identified by STOPP in 13 clients (48% of the study population)<br>**3.)** Apart from the START and STOPP criteria, the pharmacist and the investigator identified 2 potentially appropriate in dicated drugs, 81 potentially inappropriate or unnecessary drugs, 6 drug-use problems, 15	**Funding –** Nil declared.<br>**Conflict of Interest –** Nil declared.<br>**Acknowledgements -** Nil declared.

				were partially or completely implemented.	monitoring issues and 1 drug discrepancy.<br>**4.)** Mean time investment per review was 130 minutes for the investigator and 90 minutes for the pharmacist	

For Peer Review Only
